# Longitudinal Functional Study of Murine Aging: A Resource for Future Study Designs

**DOI:** 10.1002/jbm4.10466

**Published:** 2021-02-16

**Authors:** Daniel S Evans, Monique N O'Leary, Ryan Murphy, Minna Schmidt, Kristin Koenig, Michael Presley, Brittany Garrett, Ha‐Neui Kim, Li Han, Emmeline C Academia, Matt J Laye, Daniel Edgar, Christopher A Zambataro, Tracey Barhydt, Colleen M Dewey, Jarrott Mayfield, Joy Wilson, Silvestre Alavez, Mark Lucanic, Brian K Kennedy, Maria Almeida, Julie K Andersen, Pankaj Kapahi, Gordon J Lithgow, Simon Melov

**Affiliations:** ^1^ California Pacific Medical Center Research Institute San Francisco CA USA; ^2^ The Buck Institute for Research on Aging Novato CA USA; ^3^ University of Arkansas for Medical Sciences Little Rock AR USA; ^4^ Gerostate Alpha Novato CA USA

**Keywords:** AGING, BONE, MOLECULAR COMPUTED TOMOGRAPHY (mCT), OSTEOBLASTS, OSTEOCLASTS

## Abstract

Aging is characterized by systemic declines in tissue and organ functions. Interventions that slow these declines represent promising therapeutics to protect against age‐related disease and improve the quality of life. In this study, several interventions associated with lifespan extension in invertebrates or improvement of age‐related disease were tested in mouse models to determine if they were effective in slowing tissue aging in a broad spectrum of functional assays. Benzoxazole, which extends the lifespan of *Caenorhabditis elegans*, slowed age‐related femoral bone loss in mice. Rates of change were established for clinically significant parameters in untreated mice, including kyphosis, blood glucose, body composition, activity, metabolic measures, and detailed parameters of skeletal aging in bone. These findings have implications for the study of preclinical physiological aging and therapies targeting aging. Finally, an online application was created that includes the calculated rates of change and that enables power and variance to be calculated for many clinically important metrics of aging with an emphasis on bone. This resource will help in future study designs employing novel interventions in aging mice. © 2021 The Authors. *JBMR Plus* published by Wiley Periodicals LLC. on behalf of American Society for Bone and Mineral Research.

## Introduction

Many genetic and pharmacological interventions have been evaluated for lifespan extension in invertebrate and mouse models of aging,^(^ ^1^, ^2^, ^3^, ^4^, ^5^ ^)^ but few studies have focused on tissue function at advanced ages by nonsubjective criteria.^(^ ^6^, ^7^ ^)^ Tests of drugs for lifespan extension in humans are challenging because of the length of the studies. Thus, tissue and organ functions are arguably the most relevant metrics for preclinical translational studies of aging interventions.^(^ ^8^, ^9^ ^)^

Expanded translationally relevant studies of aging are urgently needed to evaluate candidate interventions for enhancing function at late ages in preclinical mammalian models.^(^ ^10^ ^)^ Those assays should be robust, objective, sensitive, relevant to human health, and noninvasive to permit longitudinal analysis throughout late life.^(^ ^8^ ^)^ For example, gait speed declines with age in humans and mice. Several efforts have attempted to develop composite indexes of aging in mice,^(^ ^8^, ^11^, ^12^ ^)^ similar to methods to incorporate multiple comorbidities under the term frailty in aged humans.^(^ ^13^, ^14^ ^)^ The World Health Organization recently created a working definition of frailty, but it may yet be modified as methods for assessing function in the elderly evolve.^(^ ^13^ ^)^

In contrast, there is universal agreement for some measures of the functional decline of many tissues and systems, such as bone, cardiovascular function, or respiratory capacity. For many of these, function can be assessed with noninvasive technology (e.g., ultrasound or CT) and monitored as individuals age. Millions of such data points exist, and we have a good understanding of individual resilience to aging based on such metrics.

In mice, the story is far less clear. Nonobjective criteria are commonly used to evaluate health outcomes in distinct tissues, instead of absolute measures (e.g., kyphosis^(^ ^6^ ^)^). In addition, variance and power are infrequently reported for such age‐related metrics in mice, making it difficult to evaluate any intervention.

**Fig 1:**
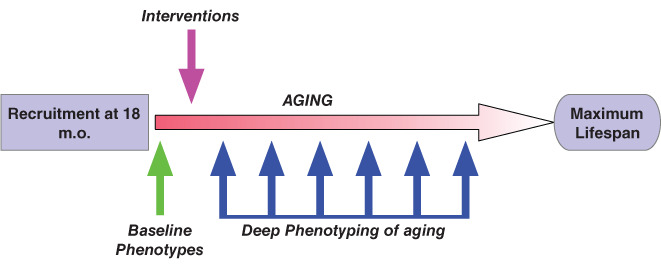
Overall study design. Recruitment of aged mice was at 18 months of age, with baseline for functional assessment established between 18–19 months of age (green arrow). At 19 months, animals were switched to a diet containing one of the four interventions (pink arrow), and were maintained on this diet for the rest of their lives. Regular functional assessments (deep phenotyping; dark blue arrows) were then carried out on individual cohorts of treated and untreated animals to facilitate detection of potential functional consequences for each intervention, approximately every quarter. Nontreated controls were also used to determine the efficacy of each intervention. We carried out functional assessments of “healthspan” using metabolic cages, cardiovascular health (echocardiography), body composition, BW, overall activity, and bone health (see Supplementary Tables S28 and S30 for list of phenotypes assayed). m.o. = months.

To address gaps in our knowledge, we initiated a “deep‐phenotyping” study of functional late‐life intervention testing in mice, emphasizing bone based on the robustness of μCT.^(^ ^15^ ^)^ We tested candidate interventions for lifespan extension, and importantly, improved tissue health.^(^ ^8^, ^9^ ^)^ We selected four interventions based on lifespan extension or disease prevention in invertebrate or mouse models: β sito‐sterol [sterol (17‐(5‐ethyl‐6‐methylheptan‐2‐yl)‐10,13‐dimethyl‐2,3,4,7,8,9,11,12,14,15,16,17‐dodecahydro‐1H‐cyclopenta[a]phenanthren‐3‐ol; BSS], clioquinol (5‐chloro‐7‐iodo‐quinolin‐8‐ol; CQ), lithium (Li), and HBX [2‐(2‐hydroxyphenyl) benzoxazole]. Overall, our strategy was to conduct noninvasive longitudinal measures of health in mice to assess metrics of functional significance and translational relevance (Fig. 1). We used mCT (micro CT) to examine the femurs of aging animals.^(^ ^16^ ^)^ Using nonsubjective criteria, we established, for the first time, the rate of change of kyphosis in aging populations of mice, determining potential benefits of each intervention on spine structure. We also established rates of change with age for control (untreated) animals (as well as cross‐sectional measures of aging vs young animals) for body composition, blood glucose, activity levels (day vs night), sleep, various respiratory measures, and body weight (BW). Finally, we established variance and subsequent power analysis for our metrics, which can be accessed via a dynamic website. These data will facilitate future studies on therapies targeting aging pathophysiology.

## Materials and Methods

### Mice

All animal procedures were carried out under an approved IACUC protocol (SM‐#A10040). We purchased 720 C57BL/6J mice aged 18 months of age from the aged colony resource of the National Institute of Aging (https://www.nia.nih.gov/research/dab/aged-rodent-colonies-handbook/eligibility-criteria-use-nia-aged-rodent-colonies) and 50 mice (aged 5 months or 155 days). C57BL/6J mice typically have mean lifespans of 26–30 months of age. At 18 months of age, mice are considered middle‐aged, and we administered our interventions in the chow beginning at approximately 19 months of age (allowing several weeks for acclimation).

### Interventions

BSS is a plant‐derived sterol reported to lower plasma cholesterol in humans.^(^ ^17^ ^)^ It has low toxicity, robust lifespan extension in *Drosophila* (Supplementary Fig. S7), and oral bioavailability.

CQ is a quinolone and antibiotic, used as an antiparasitic agent worldwide for over 500 million patient days from the 1950s to early 1970s. It was withdrawn from use because of suspected involvement in a rare neurological disorder in Japan.^(^ ^18^ ^)^ In the 2000s, it was repurposed as an antiamyloid agent in mice and is efficacious in animal models of Alzheimer disease^(^ ^19^ ^)^ and Parkinson disease.^(^ ^20^ ^)^ An analogue (PBT2) rapidly reverses age‐related cognitive defects in mice 22 months of age.^(^ ^21^ ^)^

Li (lithium carbonate) expands lifespan in *Caenorhabditis* (*C*.) *elegans* ^(^ ^22^ ^)^ and may have therapeutic benefits in Alzheimer disease and Parkinson disease.^(^ ^23^ ^)^ Li is relatively safe for humans at lower therapeutic doses.^(^ ^23^ ^)^ It is orally bioavailable, readily crosses the blood–brain barrier, and has low mutagenic and carcinogenic rates.

HBX is a synthetic molecule that binds amyloid and chelates metals. Its design was based on the amyloid dye thioflavin T (ThT).^(^ ^24^ ^)^ We found that ThT and HBX promoted protein homeostasis and extended lifespan in *C. elegans*.^(^ ^25^ ^)^ ThT promotes longevity in diverse invertebrate species.^(^ ^26^ ^)^ HBX also has drug‐like properties and low toxicity in vertebrate cell and tissue cultures.^(^ ^27^ ^)^

### Doses

We determined doses (Supplementary Table S35) based on our past work^(^ ^19^, ^28^, ^29^ ^)^ and by extrapolations from efficacious use in human disease where available. Interventions were delivered to mice from each cohort in chow fed *ad libitum*. Interventions were formulated in the chow (rodent diet 2018) by Harlan/Teklad Research Diets (Supplementary Table S35). For Li, lithium chloride was initially used but was exchanged for lithium carbonate after several days, after a washout of approximately 4 weeks caused by excessive drinking in a subset of mice on lithium chloride.

### Overall experimental design and analysis

Pre‐aged C57BL6J mice (720 mice, 18 months of age) were purchased from the National Institute on Aging (Supplementary Table S36). Before starting the experiment, 43 mice were removed because of ill health or death. Therefore, 677 mice remained up to a maximum survival of 36 months. Most of our assays had approximately 20 individuals per timepoint (from ~120 animals per intervention: 60 males and 60 females).

Using previous evaluations of variance in lifespan, we determined that we would need at least 60 animals per sex to detect a 10% difference in survival in inbred C57BL/6J mice from treatment with each of our interventions. This agrees with prior studies of lifespan.^(^ ^30^ ^)^ In addition, we repeatedly measured multiple phenotypes in a longitudinal study of a random subset of animals (~20 animals/treatment/sex) for metrics directly related to human aging.

For some assays, fewer than 20 animals were used based on limiting access of equipment at a given timepoint (e.g., metabolic cages), logistical issues with personnel, or censoring caused by death. Up to seven repeated measures were taken on approximately a quarterly basis. Staggered quarterly surveillance was carried out across the cohorts to facilitate regular functional assessment. For some timepoints, animals were added at later ages from the larger population being treated if group size decreased because of age‐related deaths. Hence, there was some “rolling recruitment” for each intervention throughout the study, which was incorporated in our overall analysis. To correct for multiple testing for the number of traits examined, the *q* value was estimated.^(^ ^31^ ^)^

### Micro‐computed tomography

Whole‐body in vivo scans were carried out on anesthetized mice (1.5% isoflurane) using a Skyscanner 1176 in vivo mCT scanner (Bruker). We used the following settings: resolution = 35 μm (image pixel size = 35.16 μm), source voltage = 50 kV, source current = 500 μA, filter Al = 0.5 mm, bit depth = 16, and frame averaging = 2. Typical numbers of individual TIFF (tag image file format) files included 1980 images generated during a scan of an individual mouse. Digital three‐dimensional (3D) reconstruction of the entire animal was done with N‐recon (Bruker), using the manufacturer's instructions with the following settings; smoothing = 2, ring artifact correction = 4, beam hardening correction = 30%, and minimum/maximum for CS to image conversion = 0–0.086987. To ensure optimal misalignment compensation during reconstruction, manual registration of the five to seven sections per animal was applied.

After reconstruction, the right femur from each animal was digitally subvolumed and saved. At each time, animals were anesthetized and scanned at 35 μm, and the subsequent skeletal data set was subvolumed to digitally extract the right femur from each animal. Log files were automatically generated during all procedures from acquisition through reconstruction and subvoluming. These log files were then used to verify that the settings used during acquisition and processing were identical for all animals to reduce the likelihood of type 1 errors. A script was written in R (R Foundation for Statistical Computing, Vienna, Austria; https://www.r-project.org/) to identify scans that varied in key parameters that could affect analysis. If errors were detected, scans were reprocessed to ensure consistency throughout the data set. After verifying that the digital data were obtained under identical conditions, the length of each femur was measured using a calibrated pixel size to calculate the midpoint of the femur. This facilitated the final targeted subvolume of 2 mm surrounding the midpoint of the diaphysis of the femur. At the resolution we used for obtaining whole‐body scans, analysis of vertebral trabecular changes with age was not possible. Therefore, we focused on changes to the femoral bone with age.

Further image processing was carried out on all samples to eliminate trabecular signal in the medullary space of the 2‐mm subvolume. Identical thresholding was carried out for each sample to delineate bone from other structures, and batch processing according to the manufacturer's instructions determined the final mean cortical metrics of bone health for the 2‐mm subvolume of femur used in the below analysis (Supplementary Fig. S8 and Table S37).

### Censored data for mCT

After eliminating animals for which there was only a single scan, a review of the 766 individuals’ remaining digitally reconstructed femurs revealed additional animals to censor. We excluded another seven mice and their repeated measures from the final data set because we determined that two of these aged mice had an abnormal bone structure (likely congenital) that required them to be excluded from the final analysis. We identified five spontaneous age‐related fractures in the right femur. Once a fracture was identified, that immediate timepoint and subsequent timepoints from the specific animal were excluded. Therefore, the final data set was 751 distinct scans, two or more times on 211 distinct individual mice.

### Other phenotypes measured

#### Metabolic and activity measures

The following metrics were assayed every quarter; overnight fasted blood glucose levels (BG), body composition (lean body mass [LBM] vs fat body mass [FBM] via an EchoMRI body composition analyzer—quantitative magnetic resonance [QMR] values), a variety of metabolic measures collected by metabolic cages, including average energy expenditure (EE), VO_2_, VCO_2_, respiratory quotient (RQ), food consumed while in metabolic cage (F), meters run on a wheel in the metabolic cage (wheel meters [WMs], day vs night), wheel speed in metabolic cage (WS; day vs night), pedestrian floor distance in metabolic cage (PM; day vs night), and sleep in metabolic cages (S; day vs night). All metabolic measures were obtained via a promethium metabolic cage system (16 cages) by Sable Systems. Measures were averaged over the second and third days or nights (2 days or 2 nights) with the initial day/night being used for acclimation to single housing (not recorded). For metabolic measures, group sizes were typically 10 animals per treatment/timepoint. Body weight (BW) was also routinely collected every 2 weeks throughout the study, representing 14,958 measures of 677 mice enrolled.

#### Mid‐diaphysis metrics of the right femur (cortical bone)

At 19 months of age, we randomly chose 10 male and 10 female mice from each treatment group that had been baselined for mCT for longitudinal μCT for each treatment, and 80 additional nontreated control mice of each sex. These mice were regularly scanned, approximately every 3 months until end of life. At later ages, additional mice from each respective treatment cohort were added to increase the numbers of surveyed mice caused by deaths in each cohort. A repeated measures design requires a minimum of two measures; so for longitudinal analysis, mice that were only scanned once were excluded from the final analysis. All measures were obtained from a subvolumed 2‐mm region of the mid‐diaphysis of the right femur, calculated by measuring the midpoint of distance from the proximal to the distal ends of the femur (Supplementary Fig. S8). In total, we carried out 751 whole‐body mCT scans on adult mice (5–36 months of age) at a resolution of 35 μm. Metrics describing mCT measurements were consistent with established guidelines.^(^ ^15^ ^)^

These 751 scans were distributed over the following group sizes: 50 untreated males, 39 untreated females (*N* = 89 controls); HBX: 14 males, 14 females (*N* = 28 HBX‐treated); CQ: 18 males, 16 females (*N* = 34 CQ‐treated); BSS: 18 males, 13 females (*N* = 31 BSS‐treated); Li: 12 males, 13 females (*N* = 25 Li‐treated); and untreated young controls: 25 males, 25 females (*N* = 50 young controls). For a full listing of metrics evaluated at the mid‐diaphysis of the femur, see Supplementary Table S37. As trabecular bone in the mid‐diaphysis is essentially absent in long bones of older animals,^(^ ^16^ ^)^ trabecular bone could not be measured. From whole‐body 35‐μm scans, we subvolumed the right femur from mice at baseline (19 months; before any intervention, *N* = 64; for initial comparison with young mice, *N* = 50).

#### Tortuosity as a measure of kyphosis (spine)

Kyphosis in mice has been measured by subjective comparative image analysis (curved vs noncurved spine via X‐ray comparisons) or a 2D kyphotic index,^(^ ^6^, ^32^ ^)^ which does not fully account for the 3D structure of the spine. Coronary tortuosity is a 3D method for analyzing the shape of arteries in multiple dimensions.^(^ ^33^ ^)^ We applied this method to the changing shape of the spine with age (Supplementary Fig. S9). Using an approach based on tortuosity, we mathematically defined the degree of curvature of the spine between two landmarks: C1 and L1 vertebrae. The 3D volumes of the spine were digitally extracted from whole‐body CT scans at 35 μm, and digital models of the spine reflecting the changing shape of the neural canal throughout the spine were constructed using a workflow developed using the 3‐matic and mimics‐research software (V20; Materialize). Tortuosity was calculated between the C1 and L2 vertebrae of the digital spine models using 1 – linear distance between C1 and L2/distance along the spine.

### Osteoclast differentiation and resorption assay

Bone marrow–derived macrophages (BMMs) were obtained as described^(^ ^34^ ^)^ from 3–4 young C57BL/6 female mice at 2–4 months of age. Whole bone–marrow cells were flushed from femurs and tibias, depleted of red blood cells with ammonium–chloride–potassium (ACK) buffer, and plated in α modified essential medium (10% FBS, 100‐U/ml^−1^ penicillin, and 100‐μg/ml^−1^ streptomycin) with macrophage colony‐stimulating factor (M‐CSF; 10 ng/ml; R&D Systems). After 24 h, nonadherent cells were replated in Petri dishes with M‐CSF (30 ng/ml) for 4 days to obtain BMMs, which were used as osteoclast precursors. To generate preosteoclasts and mature osteoclasts, BMMs were cultured in α modified essential medium with M‐CSF (30 ng/ml) and RANKL (30 ng/ml; R&D Systems) for 5 days with or without HBX. Osteoclasts were fixed with 10% neutral buffered formalin for 10 min and stained for tartrate‐resistant acid phosphatase (TRAP), using the Leukocyte Acid Phosphatase Assay Kit (Sigma‐Aldrich), following the manufacturer's instructions. A preosteoclast was defined as a round mononuclear TRAP‐positive cell and an osteoclast as a multinucleated (>3 nuclei) TRAP‐positive cell. Cells were plated in triplicate for all TRAP‐staining assays.

To investigate the effects of HBX on bone resorption in vitro, BMMs were isolated as above and stimulated with RANKL to form osteoclasts on Osteo Assay Surface 24‐well plates (Corning Life Sciences), which had an inorganic bone biomaterial surface. Cells were removed using a 2% hypochlorite solution for 5 min, washed with distilled water, and dried at room temperature. For Von Kossa staining, wells were treated in darkness with 150 μl/well of 5% (w/v) aqueous silver nitrate solution for 20 min. Plates were then washed for 5 min with distilled water and incubated in darkness with 150 μl/well of 5% (w/v) sodium carbonate in 10% formalin solution. Wells were then washed twice with PBS, rinsed with distilled water, and dried in a 50°C for 30 min. Each well was then imaged with a microscope. The resorbed areas were white, and the unresorbed mineralized surface turned black. Three wells were assessed for each group.

### Osteoblast differentiation

Calvaria cells were isolated from 3‐ to 4‐day‐old C57BL/6 mice as described.^(^ ^35^ ^)^ To obtain bone marrow–derived stromal cells, total bone marrow cells pooled from 3–4 C57BL/6 female mice at 2–4 months of age were cultured with 20% FBS, 1% penicillin–streptomycin–glutamine, and 50 μg/ml of ascorbic acid (Sigma) in 10‐cm culture dishes for 5 days. Half of the medium was replaced every 3 days. Cells were then cultured with 10% FBS, 1% penicillin–streptomycin–glutamine, 50 μg/ml of ascorbic acid, and 10mM β‐glycerophosphate (Sigma0Aldrich) for 21 days with or without HBX. Mineralized matrix was stained with 40mM Alizarin Red solution (Sigma‐Aldrich). BrdU incorporation was measured with a Cell Proliferation ELISA Chemiluminescence Kit (Roche Diagnostics). Alkaline phosphatase activity was determined in cells cultured for 7 days, and cells were lysed in 100mM glycine, 1mM MgCl_2_, and 1% Triton X‐100 at pH 10 using a buffer containing 2‐amino‐2‐methylpropanol and *p*‐nitrophenylphosphate (Sigma‐Aldrich). Alkaline phosphatase activity was normalized to total protein concentration, which was measured using a detergent‐compatible kit (Bio‐Rad). For all assays, cells were plated in triplicate.

### Statistical analysis

#### Survival

Treatment effects on survival were evaluated using Cox proportional hazards models. Parameter estimates were obtained using the following model:

$$\lambda \left(t|x\right)=\lambda_{0}\left(t\right)*\exp\;\left(\beta_{1}*\mathrm{sex}+\beta_{2}*\text{Treatment}\right)$$

*λ*(*t*| *x*) represents the hazard of death as a function of follow‐up time, conditional on the covariates sex and treatment. *λ*_0_(*t*) represents the baseline hazard. *β*_1_ represents the effect of sex on the hazard, and β_2_ represents the effect of drug treatment on the hazard. Separate models were fit for each drug treatment. The null hypothesis was β_2_ = 0. There was no evidence of violation of proportional hazards for the drug treatments as assessed by fitting an interaction term between β_2_ and follow‐up time. A *p* value ≤0.05 was deemed significant.

#### Cross‐sectional analysis

Differences in bone measures were assessed using unpaired *t* tests, as the mice at 5 and 19 months were not the same mice. A *p* value ≤0.05 was deemed significant.

#### Longitudinal analysis

Longitudinal analysis was performed using mixed effects regression models. The following mouse‐specific model was fit for each measure as an outcome:

$$y_{\mathrm{ij}}=\beta_{0}+\beta_{1}*x_{i1}+\beta_{2}*x_{i2}+\beta_{3}*x_{\mathrm{ij}3}+\beta_{4}*x_{i2}*x_{\mathrm{ij}3}+b_{i0}+b_{i3}*x_{\mathrm{ij}3}+\varepsilon_{\mathrm{ij}.}$$

where mouse *i* = 1,…, M; Observation *j* = 1,…, *n*_*i*_; *y*_*ij*_ *=* value of *j*th observation on *i*th mouse; *x*_*i*1_ = sex of the *i*th mouse; *β*_1_ = fixed‐effect parameter estimate of the sex effect; *x*_*i*2_ = intervention label of the *i*th mouse; *β*_2_ = fixed‐effect parameter estimate of the intervention effect; *x*_*ij*3_ = age of the *i*th mouse at observation *j*; *β*_3_= fixed‐effect parameter estimate of the change in bone measure over time; *β*_4_ for the interaction term (*x*_*i*2_ * *x*_*ij*3_) represented the intervention effect on change in measure over time. *b*_*i*0_ = random effects for the intercepts, and *b*_*i*3_ = random effects for the changes in measure over time (slope). *ε*_*ij*_ = error term for the mouse‐specific residuals.

Plots were created using fitted values from the model above, but with additional squared and cubed terms for mouse age and interactions with intervention for the higher‐order age terms.

Analysis of continuous traits was carried out using linear mixed‐effect regression models in R, which considered variation in group sizes with time, as well as between and within subject variance with respect to the untreated controls versus each intervention. A *p* value ≤0.05 was deemed significant. All analyses were performed using R v3.4.2. Mixed effects models were fitted using the lme4 package. Degrees of freedom were estimated using the Kenward–Roger approximation from the pbkrtest package. Test statistics were evaluated on a *t* distribution.

## Results

### Shiny app website for dynamically presenting data on aging phenotypes and interventions used herein

Our study was large (*N* > 700 animals), and we wanted to leverage our repeated measures experimental design to enable future studies to benefit from our findings regarding age‐related phenotypes. Many phenotypes were assessed in our study; so data analysis was unwieldy. Referring to dozens of tables discourages readers from effectively utilizing the collected phenotypic data on aging to assist in planning their own experiments based on our results. Therefore, we created a dynamic, online web portal for determining potential sample sizes for the age‐related phenotypes we found: https://www.danielevanslab.shinyapps.io/buckMouseAging/. This web tool will assist investigators who are planning interventional studies of aging focused on the phenotypes we measured.

Our results on age‐related trajectories and sample‐size calculations are organized hierarchically by data set, result type, and intervention group: All of which are selectable through drop‐down menus. Users begin by selecting one of the five data sets (femur mCT, kyphosis, metabolic phenotypes, survival, and weight), followed by selecting the desired result type for the data set (aging trajectories or sample‐size calculations). Sample‐size calculations are available for variables collected in the femur or metabolic data sets only. If the aging trajectories choice is selected, users can select results from control mice or different treatment groups. If controls are selected, users can view an interactive, searchable, sortable table that displays the age‐associated change parameters estimated from mixed‐effect models, whether the parameters differ by sex (PvalSexInt column), and the sex‐specific results. The table of estimates from controls provides users with an easy way to identify which measures change with age. If an intervention is selected, the treatment effect on the age‐associated trajectories of the measures from the selected data set are provided for sex combined and adjusted analyses (both), and sex‐specific analyses.

Upon selection of sample‐size calculations, users are provided a table for the selected trait with the number of mice in each group, assuming a two‐group comparison, needed to achieve 80% power to detect a particular effect size with 3–9 repeated observations based on the results from animals within this study. Sample‐size calculations are based on slope and variability parameters estimated from our mixed‐effect models. The effect size represents the percent difference in age‐associated changes for a particular trait. For a particular effect size, the sample size needed to achieve 80% power is smaller for traits that significantly change with age compared with traits that do not. Upon selecting any of the values in a sample‐size table, text is provided below the table that describes the sample‐size calculation. These sample‐size tables can be used to prioritize traits to examine in future studies of aging in mice, as well as to aid in the study design related to the number of mice and number of repeated measures to perform.

The complete set of results and the code to generate the web application are downloadable from the GitHub repository linked within the “Results and Code” tab of the website. We also provide extensive Supplementary tables allowing offline analysis of our results, which are linked to this article. Throughout this article, when we refer to the shiny app implemented via Github, we use the term Phenotype website.

### Effect on overall survival for each intervention

In our study, we determined the effects of the interventions on lifespan. In sex‐adjusted analyses, no intervention was associated with survival (Supplementary Fig. S1). In sex‐stratified analyses, CQ was associated with a higher mortality risk in males (hazard ratio [HR] = 1.50). HBX was nonsignificantly associated with a lower mortality risk (HR = 0.83) in sex‐adjusted analyses, consistent with a potential beneficial effect. A more‐comprehensive experimental design with multiple doses may uncover beneficial effects on survival for one or more interventions. In a study with HBX looking at survival alone via the intervention testing program, mean lifespan was not extended,^(^ ^36^ ^)^ although median lifespan was higher in males, consistent with the results here.

### Cross‐sectional analysis of aging bone microarchitecture

The microarchitecture of aging mouse bone shows dynamic changes, with reported outcomes similar to the aging human skeleton.^(^ ^37^, ^38^, ^39^ ^)^ Long bones, such as the femur, are composed of trabecular and cortical bone. Most trabecular bone has been resorbed in long bones of C57BL/6J mice over 6 months of age, particularly in females. Therefore, we focused on the cortical bone of the midshaft of right leg femurs, as μCT provides high contrast in this area and trabecular bone is essentially absent.^(^ ^16^ ^)^ We acquired images at 35‐μm resolution. At this level, trabecular bone is not adequately resolved, particularly at other sites, such as vertebra.^(^ ^15^ ^)^ Cortical bone has less structural variation over time than other parts of the skeleton.

As in published studies, we identified many significant differences in bone microarchitecture in a cross‐sectional comparison of young and middle‐aged mice (Table 1). The two age groups in both sexes differ in a number of important metrics of bone aging, including bone volume/total volume (BV/TV), bone surface area (BS), periosteal perimeter (B.Pm), eccentricity, cortical thickness), endosteal perimeter, and medullary area (Table 1). By 19 months of age (“middle‐aged” = ~70% of mean lifespan = 27 months or approximately 55 years in humans), many degenerative changes have already taken place in the femur in agreement with other cross‐sectional studies.^(^ ^16^ ^)^ These included a decrease in BV, thinning of the cortices of the femur, expansion of the cortical envelopes (periosteal and endosteal), and an increase in the marrow cavity (medullary area), similar to reports of age‐related changes in cortical bone from middle‐aged mice.^(^ ^16^, ^37^, ^38^, ^39^, ^40^ ^)^

**Table 1:** Age‐Related Metrics for Mid‐Diaphysis Femoral Cortical Bone

Phenotype	n_total^a^	ß^b^	SE	*p*	*n*_5 mo^c^	*n*_19 mo^d^
TV	114	0.877	0.072	3.1E‐22	50	64
BV	114	0.148	0.037	1.2E‐04	50	64
BV.TV	114	‐5.164	0.430	8.8E‐22	50	64
TS	114	1.842	0.178	6.1E‐18	50	64
TS.per	114	1.069	0.096	7.9E‐20	50	64
BS	114	2.882	0.386	1.9E‐11	50	64
BS.per	114	2.464	0.268	2.8E‐15	50	64
BS.BV	114	0.482	0.145	1.2E‐03	50	64
T.Ar	114	0.351	0.029	3.1E‐22	50	64
T.Pm	114	0.428	0.038	7.9E‐20	50	64
B.Ar	114	0.059	0.015	1.2E‐04	50	64
B.Pm	114	0.987	0.107	2.8E‐15	50	64
Ecc	114	‐0.048	0.005	1.7E‐17	50	64
Cs.Th	114	‐0.007	0.002	2.2E‐03	50	64
Po.cl	114	4.712	0.556	1.1E‐13	50	64
endosteal	114	0.559	0.078	7.9E‐11	50	64
Med.area	114	0.292	0.019	7.1E‐29	50	64

B.Ar = mean total cross‐sectional bone area; B.Pm = mean total cross‐sectional bone perimeter; BS = bone surface; BS.BV = bone surface/volume ratio; BS.per = bone surface percent; BV = bone volume; BV.TV = percent bone volume; Cs.Th = cross‐sectional thickness; Ecc = eccentricity; endosteal = endosteal perimeter; Med.area = medullary area; Po.cl = closed porosity; SE = standard error of the mean; T.Ar = mean total cross‐sectional tissue area; T.Pm = mean total cross‐sectional tissue perimeter; TS = tissue surface; TS.per = tissue surface percent; TV = total volume. ^a^ *n*_total = total number of animals measured. ^b^ß = calculated mean outcome of older animal phenotype vs younger. ^c^ *n*_5months = number of animals at 5 mo of age used for comparison. ^d^ *n*_19months = number of animals at 19 mo of age used for comparison.

For the first time, we report that variability increased between 5 and 19 months for many of the measured metrics in the aging femur (Phenotype website, and Supplementary Table S1). Somewhat surprisingly, these phenotypes became less variable as animals grew older (TV, tissue surface, tissue surface percent, BS, BS percent, mean total cross‐sectional tissue area, mean total cross‐sectional tissue perimeter, and B.Pm; *p* < 0.001). Several traits were different at the mean, but variance was unchanged with age (percent bone volume [BV.TV], closed porosity). The variability we report is observed in genetically identical mice; hence, the source of this age‐related variability is unlikely to be mediated solely through allelic variation.

**Fig 2:**
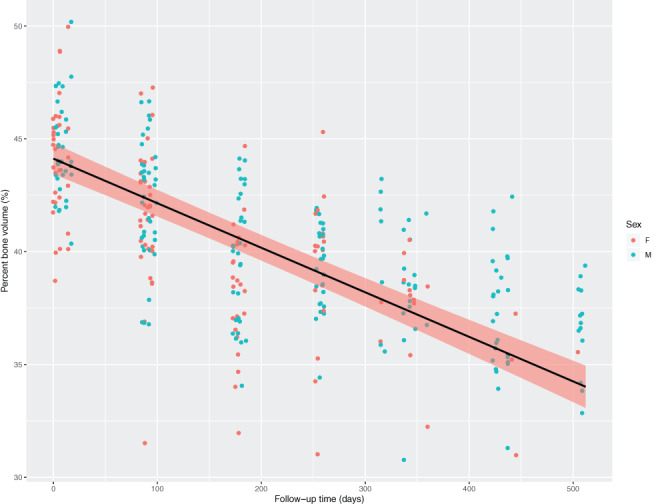
Bone volume changes of the midfemur in late life. A linear model for the rate of change of loss of mid‐diaphysis for untreated animals (*N* = 89) was calculated. ß represents the calculated outcome of the percentage of bone volume with increasing age, resulting in a 1.94% loss in bone volume per 100 days on average after adjusting for sex. Sex‐specific effects were noted (*p* = 0.008), with males (*N* = 48) having a reduced ß in this age range of 0.009 (*p* = 0.0002) versus a ß of 0.02 for females (*N* = 41; *p* = 7.04).

Having confirmed the presence of significant differences by midlife (19 months old) via cross‐sectional analysis, we next determined which skeletal parameters further changed over the remainder of the animals' lives through a repeated measures approach using a mixed‐effect linear model. This approach permitted the calculation of the rate of change with age for each of the metrics presented (Table 2). After adjusting for sex, BV is lost in the midfemoral shaft at a rate of 1.94% every 100 days between 19 and 36 months of age (Fig. 2, Table 2). This age range approximates the greatest changes in bone health with human aging. The Phenotype website and Supplementary Tables S2 and S3 describe sex‐specific differences with advanced age for femoral mid‐diaphysis of cortical bone.

**Table 2:** Age‐Related Metrics for Mid‐Diaphysis Femoral Cortical Bone in 19‐ to 36‐Month‐Old Untreated Animals

	*N*	ß^a^	SE	*p*	*p* (sex interaction)^b^
TV	89	0.171	0.009	2.2E‐33	7.5E‐06
BV	89	–0.053	0.007	6.7E‐12	0.100
BV.TV	89	–1.941	0.107	2.3E‐31	0.109
TS	89	0.689	0.036	6.0E‐33	0.115
TS.per	89	0.239	0.011	2.8E‐36	9.0E‐06
BS	89	1.327	0.169	9.4E‐12	0.580
BS.per	89	0.903	0.119	2.8E‐11	0.767
BS.BV	89	0.818	0.063	1.6E‐22	0.318
T.Ar	89	0.069	0.003	2.2E‐33	7.5E‐06
T.Pm	89	0.096	0.004	2.8E‐36	9.0E‐06
B.Ar	89	–0.021	0.003	6.7E‐12	0.100
B.Pm	89	0.362	0.048	2.8E‐11	0.767
Ecc	89	–0.007	0.001	5.3E‐10	0.012
Cs.Th	89	–0.009	0.001	3.1E‐24	0.400
Po.cl	89	1.290	0.262	4.2E‐06	0.538
endosteal	89	0.259	0.044	5.5E‐08	0.494
Med.area	89	0.092	0.004	4.0E‐37	0.030

B.Ar mean total cross‐sectional bone area; B.Pm = mean total cross‐sectional bone perimeter; BS = bone surface; BS.BV = bone surface/volume ratio; BS.per = bone surface percent; BV = bone volume; BV.TV = percent bone volume; Cs.Th = cross‐sectional thickness; Ecc = eccentricity; endosteal = endosteal perimeter; Med.area = medullary area; Po.cl = closed porosity; SE = standard error of the mean; T.Ar = mean total cross‐sectional tissue area; T.Pm = mean total cross‐sectional tissue perimeter; TS = tissue surface; TS.per = tissue surface percent; TV = total volume. ^a^ß = Calculated mean outcome of phenotype per 100 days between 19 and 36 months of age; ^b^ *p* (sex interaction) = *p* < 0.05. ß is significantly different by sex.

Given the large sample size, our estimates of within and between mouse variability enabled us to perform power calculations to facilitate sample‐size estimation for future studies to detect treatment effects for each listed metric. The Phenotype website and Supplementary Table S4 feature an example calculation, showing that to detect a 15% change in BV, using five repeated measures every 100 days starting at 19 months of age, one would need to enroll 50 animals in each. This approach can be generalized to other phenotypes, providing a resource for studies to detect meaningful differences with novel interventions. The Phenotype website and Supplementary Tables S4–S26 provide power analysis for many metrics of interest to the research aging community, enabling future study designs testing candidate interventions for beneficial effects on these important age‐related phenotypes.

### Spontaneous fracture frequency in aged mice

**Fig 3:**
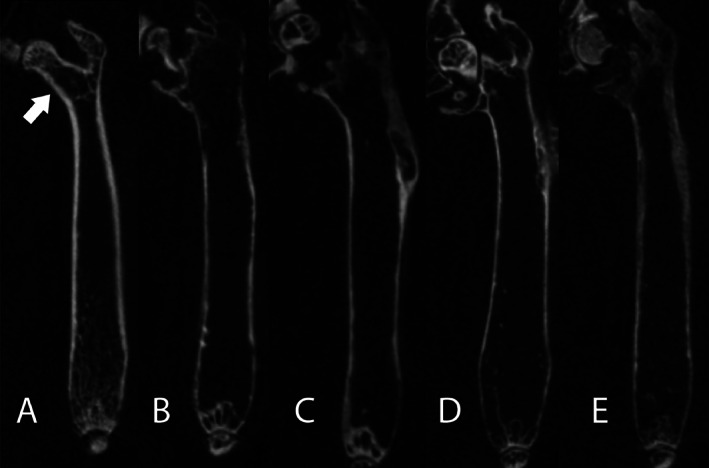
**(*A*‐*E*)**. Age‐related fractures in mice. μCT cross‐sectional images of right femurs of mice aged 5 (***A***), 25 (***B***), 27 (***C***), and 30 (***D***,***E***) months of age. Images were scanned at a resolution of 35 μm in vivo. The intact femoral neck of a young mouse femur is highlighted by an arrow in (*A*). (***B***‐***E***) images show severe fractures at the femoral neck with complete displacement.

The most serious consequences of loss of bone mass with age are fractures of the femur, some of which occur in the femoral neck,^(^ ^41^ ^)^ structurally the weakest part of the femur. Our repeated measures strategy of observing femurs in vivo from 19 to 36 months of age permitted an estimation of the rate of spontaneous breaks in the femur, all of which occurred in the femoral neck (Fig. 3). None occurred before 24 months, which may explain why this age‐related pathology has not been reported. We report frequency of fractures of the right femur of 2.6% in animals ≥24 months of age based on our observation of five fractures (two females, three males) in 191 individuals scanned from 24 months of age or older. There were no fractures in animals <24 months of age.

### HBX treatment slowed bone aging in late life and mitigated age‐related bone loss

**Fig 4:**
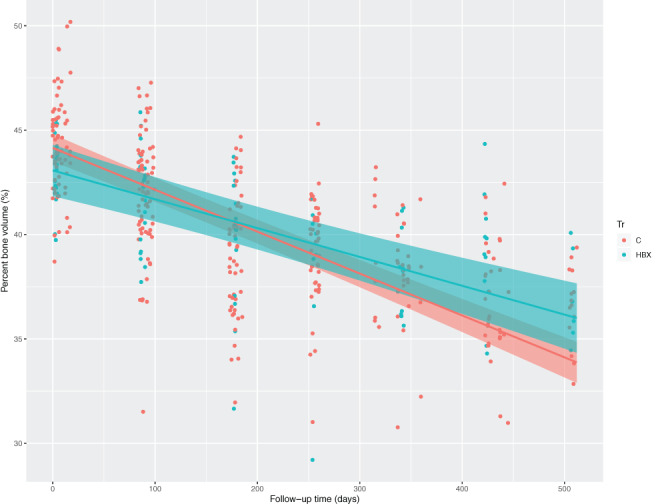
HBX [2‐(2‐hydroxyphenyl) benzoxazole] treatment slows bone aging in the cortical midshaft. Bone volume/total volume is shown as a percent on the *y*‐axis, and follow‐up time in days on the *x*‐axis. Animals treated with HBX decline slower in the rate of bone loss; 4.9% per year compared with 7.1% per year of untreated controls (*p* = 0.006). Blue = controls, red = HBX treated.

A linear model was fitted to the repeated measures (Fig. 4). HBX treatment led to a pronounced reduction in the rate of change of multiple measures of bone microarchitecture. HBX treatment partially attenuated the trajectory, indicating beneficial outcomes of treatment by HBX (Table 3): 31% of BV was spared over a year, a larger effect than that seen in humans with chronic bisphosphonate treatment over 2–3 years. Cortical thickness was 27% greater than controls, and bone cross‐sectional area was 93% greater than controls. HBX's effects on age‐related changes in BV, BV/TV, and B.Ar were significant after correction for multiple testing (*q* value < 0.05 for all three phenotypes). Sex‐specific HBX treatment effects were not observed for BV/TV. CQ and Li yielded no changes with increased age. BSS resulted in a few marginally significant changes (BV/TV), indicating potential for benefit.

**Table 3:** HBX Treatment Slows Key Metrics of Cortical Bone Aging in Late Life (19–36 Months of Age)

Phenotype	*N* (Control)	*N* (HBX)	ß^a^	SE	*p*	*p* (intersex)^b^
TV	89	28	–0.002	0.019	0.907	4.6E‐05
BV	89	28	0.039	0.014	0.007	0.299
BV.TV	89	28	0.604	0.217	0.006	0.126
TS	89	28	–0.121	0.073	0.100	0.118
TS.per	89	28	–8.6E‐06	0.023	1.000	6.4E‐05
BS	89	28	–0.095	0.337	0.778	0.410
BS.per	89	28	–0.071	0.241	0.770	0.579
BS.BV	89	28	–0.198	0.126	0.119	0.458
T.Ar	89	28	–0.001	0.008	0.907	4.6E‐05
T.Pm	89	28	–0.000	0.009	1.000	6.4E‐05
B.Ar	89	28	0.016	0.006	0.007	0.299
B.Pm	89	28	–0.028	0.096	0.770	0.579
Ecc	89	28	–0.001	0.002	0.502	0.005
Cs.Th	89	28	0.003	0.001	0.032	0.627
Po.cl	89	28	–0.921	0.579	0.115	0.872
Endosteal	89	28	–0.016	0.088	0.857	0.350
Med.area	89	28	–0.016	0.008	0.047	0.033

B.Ar = mean total cross‐sectional bone area; B.Pm = mean total crossectional bone perimeter; BS = bone surface; BS.per = bone surface percent; BS.BV = bone surface/volume ratio; BV = bone volume; BV.TV = percent bone volume; Cs.Th = cross sectional thickness; Ecc = eccentricity; endosteal = endosteal perimeter; Med.area = medullary area; Po.cl = closed porosity; SE = standard error of the mean; T.Ar = mean total cross‐sectional tissue area; T.Pm = mean total cross‐sectional tissue perimeter; TS = tissue surface; TS.per = tissue surface percent; TV = total volume. ^a^ß = Calculated mean difference per 100 days for outcome of animals treated with HBX vs controls. ^b^ *p* (intersex) = *p* < 0.05 ß is significantly different by sex.

To determine HBX's effects on cell types involved in bone loss with age, we used bone marrow–derived osteoclasts. Resorption activity in response to 5–40μM HBX was markedly impaired without affecting the osteoclast number (5–20μM; Supplementary Fig. S2). At 20–40μM, osteoclast progenitors were reduced by approximately 50%. We determined that in vitro osteoblast formation, accessed by Alizarin Red staining, was inhibited by HBX at doses higher than 5μM. Low doses (0–10μM) also inhibited osteoblast formation from stromal cells from long bones (Supplementary Fig. S3). Inhibitory effects on cell differentiation, determined by alkaline phosphatase activity, and proliferation in stromal and calvaria cells contributed to the antiosteoblastogenic actions of HBX. In vivo, our finding that HBX attenuated the loss of bone mass suggested that its effects on bone resorption may be predominant.

### Cross‐sectional and longitudinal analysis of age‐related changes

We measured multiple metabolic and body composition traits: BG, LBM, and FBM evaluated by QMR, BW assayed approximately every 2 weeks throughout late life, EE, VO_2_, VCO_2_, RQ, F, WM, WS, PM, and S (Phenotype website and Supplementary Table S27).

Cross‐sectional comparisons of 5‐ and 19‐month‐old mice revealed that 17 of the 29 metabolic phenotypes changed with age (Table 4). The ß value shows the phenotype difference in older and younger animals, and whether it is increased or decreased with age. We determined ß for outcomes that improved health. For example, an improved outcome changed a negative ß to positive.

**Table 4:** Metabolic Changes Between 5 Months of Age and 19 Months of Age

Phenotype^a^	Total *N* ^b^	ß^c^	SE	*p*	*N* (5 mo)	*N* (19 mo)
BG	97	3.084	6.371	0.630	32	65
EE.BMD	56	–0.001	3.5E‐04	0.036	32	24
EE.BMN	56	–0.004	0.001	1.8E‐07	32	24
EE.LBMD	56	–0.001	4.1E‐04	0.165	32	24
EE.LBMN	56	–0.004	0.001	2.0E‐06	32	24
EED	56	0.024	0.012	0.042	32	24
EEN	56	–0.050	0.021	0.018	32	24
FBM	97	2.273	0.377	3.3E‐08	32	65
FD	56	–0.328	0.147	0.030	32	24
FN	56	–0.224	0.224	0.322	32	24
LBM	97	1.519	0.319	6.8E‐06	32	65
PMD	56	0.922	2.882	0.750	32	24
PMN	56	–5.232	7.843	0.508	32	24
PSD	56	–0.001	5.0E‐04	0.093	32	24
PSN	56	–0.001	4.9E‐04	0.114	32	24
RQD	56	–0.142	0.021	6.7E‐09	32	24
RQN	56	–0.143	0.020	4.0E‐09	32	24
SD	56	0.093	0.429	0.830	32	24
SN	56	0.702	0.253	0.008	32	24
VCD	56	–0.100	0.048	0.043	32	24
VCN	56	–0.395	0.084	1.8E‐05	32	24
VOD	56	0.130	0.038	0.001	32	24
VON	56	–0.101	0.065	0.130	32	24
WD	56	0.055	0.102	0.594	32	24
WMD	53	–141	77	0.074	29	24
WMN	53	–3230	544	2.7E‐07	29	24
WN	56	–0.271	0.145	0.067	32	24
WSD	52	–0.034	0.014	0.022	29	23
WSN	52	–0.064	0.011	8.1E‐07	29	23

BG = fasted blood glucose level; BM = body mass; EE = energy expenditure; EE.BM = energy expenditure/body mass; EE.LBM = energy expenditure/lean body mass; F = food consumed; FBM = fat; LBM = lean body mass; PM = meters walked; PS = mean speed of walking; RQ = respiratory quotient; S = sleep; SE = standard error; VC = VCO_2_; VO = VO_2_; W = water drunk; WM = wheel meters run; WS = wheel speed while running. ^a^(Phenotype) D = average by day; (Phenotype) N = average by night. ^b^Total N = Total numbers of animals per measured phenotype are shown. ^c^ß = Change relative to young animals (adjusted for sex).

Most phenotypes were similar in this cross‐sectional comparison, but some (light gray in Table 4) were significantly different. EE and LBM at night were less in older animals than in young animals, F increased (2.273 g more in older), LBM increased with age (1.519 g more in older), RQ decreased by day and night in older, sleep by night decreased by 0.702 of an hour in older animals, VCO_2_ decreased by night/day in older animals, and VO_2_ increased by day (Table 4). Most striking but not unexpected, older animals ran less at night (<3230 m/night), and ran slower at day or night (<0.064 m/s per night for older animals). In males, eight phenotypes were significantly different; in females, 16 phenotypes were significantly different (Phenotype website and Supplementary Tables S28 and S29). All phenotypes different in males were also different in females. Female‐specific significant differences were observed for EE at night, F during day and night, S at night, VCO_2_ in the day, VO_2_ in the day, and wheel speed at day and night.

Based on these differences, we characterized a “young” metabolic physiology. We continued to evaluate these phenotypes in untreated control animals to estimate age‐related rates of change after 19 months by linear mixed‐effect models (Phenotype website and Supplementary Tables S30–S32). Adjusting for the effects of sex, 18 of the 29 phenotypes changed significantly with age from 19–36 months of age. Notable among the phenotypes being assessed, BG declined with age at a rate of 12 mg/dl on average every 100 days. All the movement‐related metrics declined, particularly the night‐related measures. Night‐time energy expenditure–related metrics and respiratory quotients also declined.

Some interventions had no effect and a few showed adverse changes. After adjusting for sex, Li had potentially deleterious effects on the animals without affecting survival. Blood glucose increases with age in humans and decrease in mice.^(^ ^42^ ^)^ CQ treatment improved blood glucose levels with age by 13 mg/dl relative to untreated controls from 19–35 months of age, after adjusting for sex (Phenotype website and Supplementary Table S33).

### Decline in body weight with age

Among the 217 controls (110 males, 107 females) used from 19–36 months of age, after adjusting for sex, BM declined with age (0.5 g lost per 100 days; Phenotype website and Supplementary Fig. S4). BM loss was significant in males (0.94 g lost per 100 days) but not females, and the difference by sex was significant. In sex‐stratified analyses of treated animals, BSS treatment in males was associated with an increase in BM with age that was larger than the BM decline in controls (0.6 g per 100 days increased over untreated controls). BSS treatment was not associated with a significant effect on BM in females. CQ treatment in males was associated with a decrease in BM with age relative to controls (−0.6 g per 100 days compared with untreated controls). CQ treatment was not associated with BM in females. BM loss or gain was not significantly different from untreated controls in Li‐treated animals. Finally, HBX treatment was not significantly associated with BW in males, females, or when sexes were combined.

### Characterization of kyphosis in aging populations of mice by mCT

Kyphosis, as measured by tortuosity, showed a clear age‐related increase (5‐ to 19‐months‐old; Supplementary Fig. S5*A*). In addition, the onset of kyphosis from 19–36 months of age is highly variable (Supplementary Fig. S5*B*). By 19 months of age, there is a change in the degree of kyphosis of the spine (Supplementary Table S34). For males and females individually, the increase in tortuosity is greater in males than females (ß values of 0.034 and 0.019, respectively). From 19–36 months of age, tortuosity varied considerably at each age (Supplementary Fig. S5). In controls, the ß value was 0.01 of a unit increase on average per 100 days (Supplementary Fig. S6). The increase in tortuosity was greater in females than males, with females having a ß of 0.02 per 100 days, versus 0.009 for males per 100 days. None of the interventions slowed the degree of tortuosity with increasing age.

## Discussion

Here we tested interventions associated with lifespan extension in invertebrates or improvement of age‐related disease in mouse models. We found that HBX slowed age‐related femoral bone loss in mice. We established rates of change for clinically significant parameters in untreated mice, including kyphosis, blood glucose, body composition, activity, metabolic measures, and detailed parameters of skeletal aging in bone. Finally, we created an online application that will help in future study designs employing novel interventions in aging mice. Our findings have implications for the study of preclinical physiological aging and therapies targeting aging.

Over the last 30 years, aging research has exploded into mainstream science.^(^ ^43^ ^)^ Aging is seen as more of a tractable biological trait that can be altered.^(^ ^4^ ^)^ Such approaches often increase lifespan and attenuate progressive physiological decline.^(^ ^3^, ^5^, ^37^, ^43^, ^44^, ^45^, ^46^, ^47^ ^)^ Despite substantive breakthroughs in geroscience, it is unclear if lifespan extension is always associated with an increase in healthy life (healthspan).^(^ ^48^ ^)^

Although many aspects of aging physiology in model systems are interesting, phenotypes directly translatable to age‐related functional decline in humans are the most relevant for rational therapeutics.^(^ ^9^ ^)^ Although invertebrate model systems are excellent for evaluating potential lifespan‐extending interventions, they are often lacking in translatable age‐related phenotypes. Conversely, though vertebrate systems are useful for evaluating translatable physiological functions, they are relatively poor for identifying compounds that extend lifespan because of a lack of throughput, expense, and length of time involved. Here we use the strengths of both vertebrate and invertebrate systems to test interventions that were successful in the extension of lifespan, for efficacy in slowing the decline of functional domains of aging, including loss of bone.

We focused on repeated measures. By capturing a single time in “old” animals, cross‐sectional approaches ignore trajectories of changing health, which is the parameter of interest in studies of human aging. A repeated measures approach is infrequently used to assess translationally relevant metrics in animal studies of aging,^(^ ^6^, ^37^, ^49^ ^)^ but is common in clinical trials because of key advantages in statistical inference with regards to within‐subject changes over time.^(^ ^50^ ^)^ For the skeleton, we used μCT. This imaging technique has advantages for evaluating functional decline of bone, including the ability to repeatedly measure the same individual over time.

A number of prior studies reported skeletal changes with age in multiple strains of mice, all of which had similarities to those observed in human subjects.^(^ ^37^, ^38^, ^40^ ^)^ However, most prior mouse studies did not go significantly beyond middle‐aged mice (19–24 months of age). C57BL/6J is the most used strain of mice for aging studies and has reported mean lifespans in the 26‐ to 30‐month range. Hence, we evaluated pathophysiological changes from mid through late life in the context of our candidate interventions. Arguably, this strategy will likely be used in human trials.

We developed a dynamic website that infers potential benefits of candidate interventions for a range of outcomes at early stages of an experimental design. This could potentially save substantial resources if the desired beneficial effect is unlikely to be detected if the proposed cohort size is too small. Our description of a new method for evaluating kyphosis using nonsubjective criteria could also be useful for evaluating novel therapeutics for this important age‐related disorder. Until now, it has been difficult to measure kyphosis with unbiased methodologies. We used μCT to measure tortuosity of the spine, and that may pave the way for testing new interventions with kyphosis.

We used an inbred strain of mouse to eliminate genetic variation. The animals had identical housing and diets. Despite identical genetics, environment, and diet, there is as much as a fivefold increase in variance with age in some metrics in the cortex of the midfemur and other metrics. The origin of the increased age‐related variation is unknown. Epigenetic modifiers are a possibility. Identifying the cause could lead to studies to determine if that variation contributes to the heterogeneity in bone mass and loss among postmenopausal women.

We also determined, for the first time, a spontaneous fracture occurrence of 2.5% not dissimilar to the 1%–2.7% incidence of hip fractures in people over the age of 65.^(^ ^51^ ^)^ Spontaneous fractures as a function of age in old mice have never been reported. The cause of fractures in the elderly is primarily falls, compounded by osteopenic changes in the skeleton. Similar trauma in caged mice is unlikely. Intriguingly, the site of fracture in aged mice is a common site for fractures in elderly humans: the neck of the femur. Our fracture data suggest new approaches to study skeletal dynamics and to determine if treatment can reduce the incidence of such fractures.

HBX decreased osteoclast resorptive activity at low doses with no impact on osteoclast formation. It inhibited osteoclast progenitor proliferation and osteoclast formation at higher doses. These findings strongly suggest that suppressing bone resorption is responsible for the bone‐sparing effects of HBX in vivo.^(^ ^52^ ^)^ The beneficial effects we report for HBX in bone cells in vitro are reminiscent of similar findings with estrogens. Although in vivo antiosteoblastogenic actions of estrogens are mostly secondary to their antiresorptive activity,^(^ ^53^ ^)^ estrogens may inhibit osteoblastogenesis.^(^ ^54^ ^)^ On balance, our results imply that the action of HBX on bone frailty with age is via an antiresorptive activity on osteoclasts, similar to the current main class of drugs used in treating osteoporosis, the bisphosphonates. A 31% reduction in bone loss late in life would be considered substantial if this was recapitulated in the aging human skeleton.

A key question uncovered by the results of our study is why does an intervention that extended lifespan in *C. elegans* slow bone loss in aging mice? The mechanism of action of HBX on lifespan extension may involve suppression of increased age‐related protein aggregation. in vitro assays of protein aggregation (using peptides, such as amyloid β in Alzheimer disease) show that HBX sequesters metal ions and dramatically slows metal‐promoted amyloid fibril formation.^(^ ^24^ ^)^ We hypothesize that misfolded proteins mediate pathology in tissues other than brain, including bone. Our success in using chemicals that promote lifespan in invertebrates to identify therapeutic interventions for age‐related pathologies is a promising strategy to combat the diseases of aging. This is consistent with prior successful lifespan extending interventions such as rapamycin.^(^ ^55^, ^56^ ^)^

Finally, the utility of leveraging our large body of aging animal data for future study designs is a key outcome of this study. We hope that investigators will avail themselves of this resource to design future interventional experiments in aging mice to discover novel potential therapeutics for age‐related disease and dysfunction.

## AUTHOR CONTRIBUTIONS

**Daniel Evans:** Formal analysis; software; writing‐original draft; writing‐review & editing. **Monique O'Leary:** Investigation. **Ryan Murphy:** Investigation. **Minna Schmidt:** Investigation. **Kristin Koenig:** Investigation. **Michael Presley:** Investigation; software. **Brittany Garrett:** Investigation. **Ha‐Neui Kim:** Investigation. **Li Han:** Investigation. **Emmeline Academia:** Investigation. **Matt Laye:** Investigation. **Daniel Edgar:** Investigation, Experimental. **Christopher Zambataro:** Investigation. **Tracey Barhydt:** Investigation. **Colleen Dewey:** Investigation. **Jarrott Mayfield:** Investigation. **Joy Wilson:** Investigation. **Silvestre Alavez:** Conceptualization. **Mark Lucanic:** Writing‐review & editing. **Brian Kennedy:** Resources. **Maria Almeida:** Investigation; validation; writing‐review & editing. **Julie Andersen:** Conceptualization. **Pankaj Kapahi:** Conceptualization; methodology; writing‐review & editing. **Gordon Lithgow:** Conceptualization; writing‐review & editing. **Simon Melov:** Conceptualization; data curation; formal analysis; investigation; methodology; supervision; visualization.

### PEER REVIEW

The peer review history for this article is available at https://publons.com/publon/10.1002/jbm4.10466.

## Supporting information


**Figure S1** Supporting information

**Table S1** Supporting information

## Data Availability

The data that support the findings of this study are openly available at https://www.danielevanslab.shinyapps.io/buckMouseAging/. The complete set of results and the code to generate the web application is downloadable from the GitHub repository linked within the “Results and Code” tab of the website.
