# Supplementary material for: Longitudinal Functional Study of Murine Aging: A Resource for Future Study Designs
Source: JBMR Plus. 2021 Feb 16;5(3):e10466. doi: 10.1002/jbm4.10466 (PMC7990142; doi:10.1002/jbm4.10466)

## Supplemental Figures, Evans et al.

***Supplemental Figure 1.*** Survival curves of 4 cohorts of mice treated with A) CQ (Clioquinol), B) Li (Lithium Carbonate), C) HBX (2-(2-hydroxyphenyl) benzoxazole, or D) BS (Beta Sitosterol) contrasted with untreated controls. No significant increase in lifespan was seen with these interventions at the dose used.

**A)**

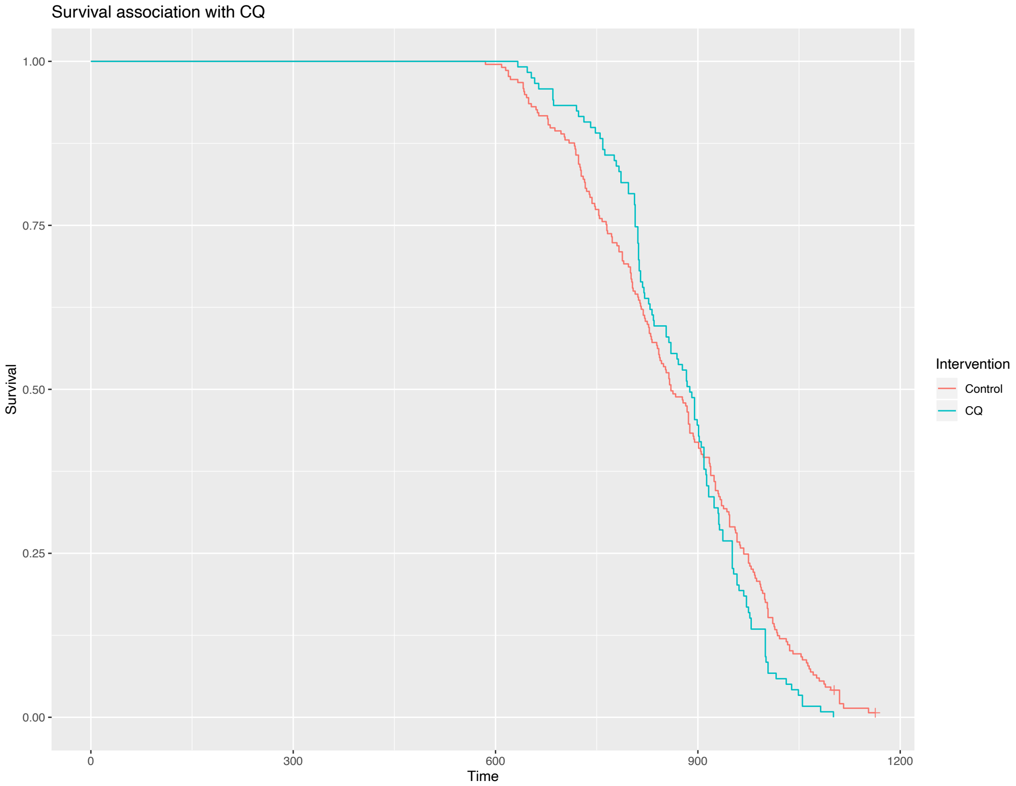


**B)**

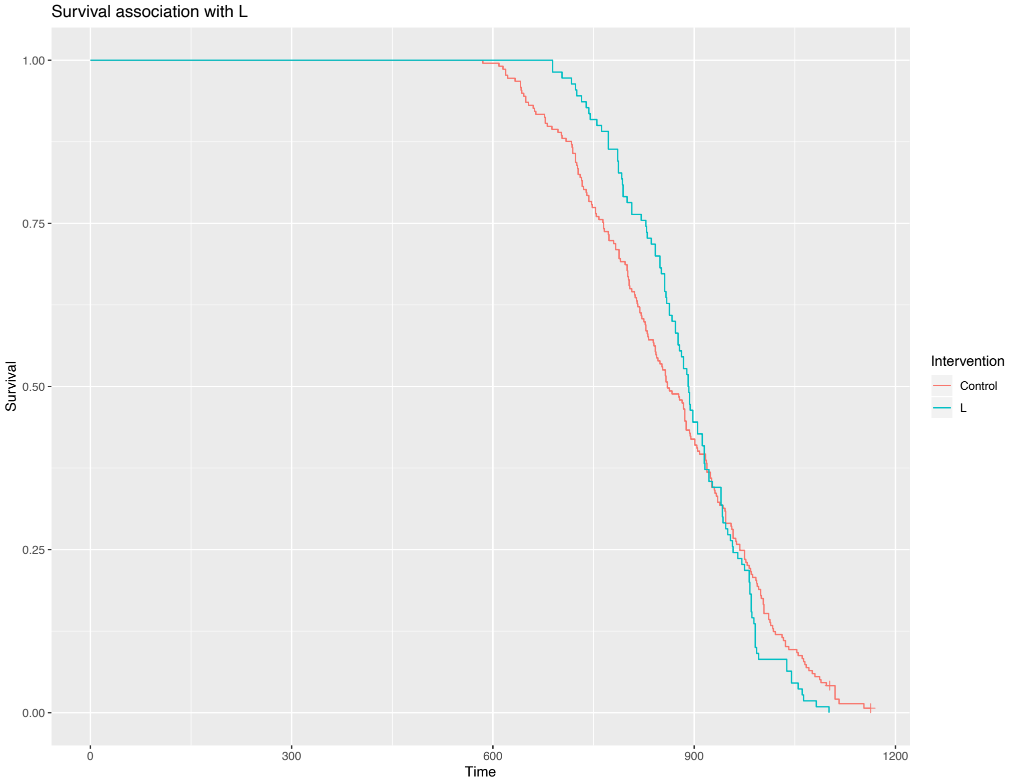

C)

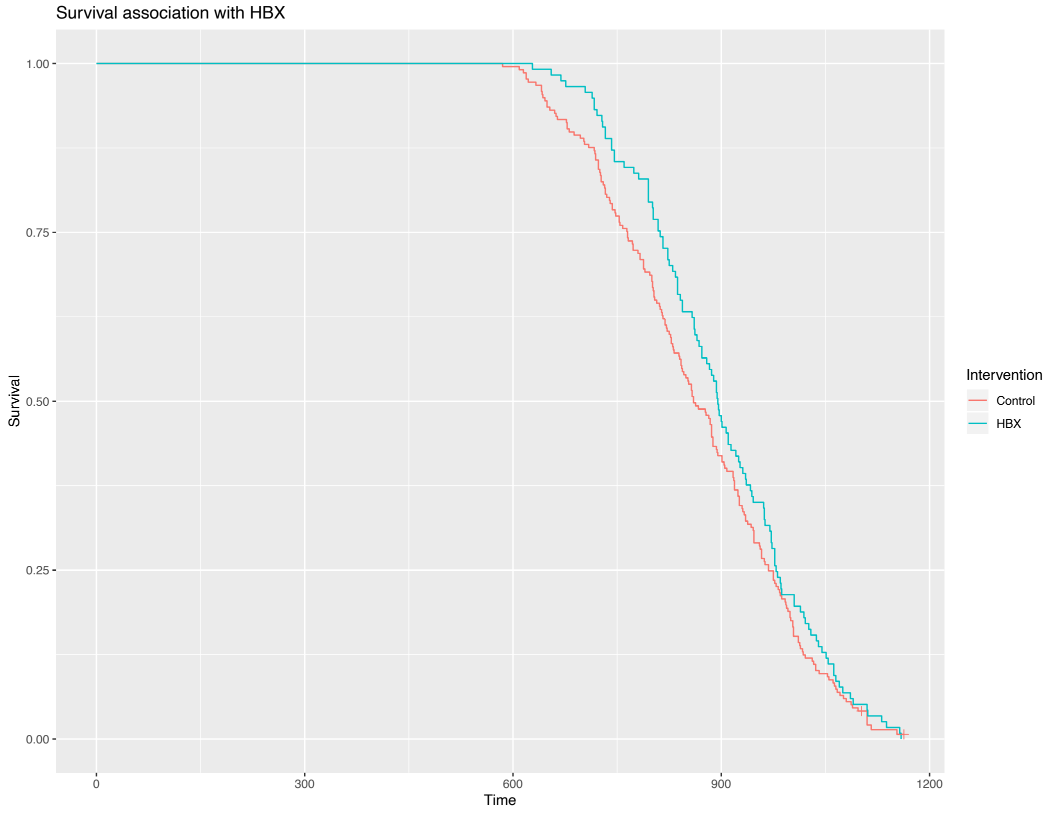
Survival association with HBX
Survival
1.00
0.75
0.50
0.25
0.00
0
300
600
900
1200
Time
Intervention
Control
HBX

D)

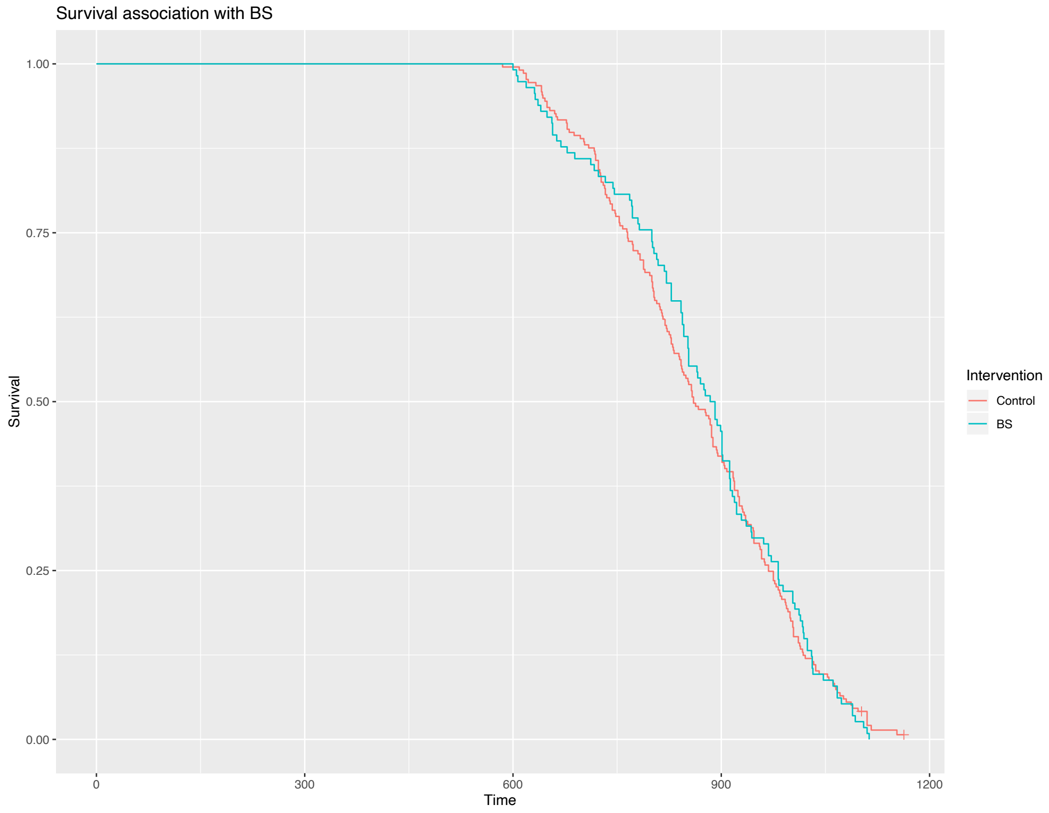
Survival association with BS
Survival
1.00
0.75
0.50
0.25
0.00
0
300
600
900
1200
Time
Intervention
Control
BS

***Supplemental Figure 2. HBX is an anti-resorbtive*****.** A) Resorption assay showing dose dependent inhibition of HBX on osteoclast activity. B) TRAP staining & Proliferation of osteoclastogenesis are inhibited without cell death by increasing dosage of HBX.

**(A) Resorption Assay**

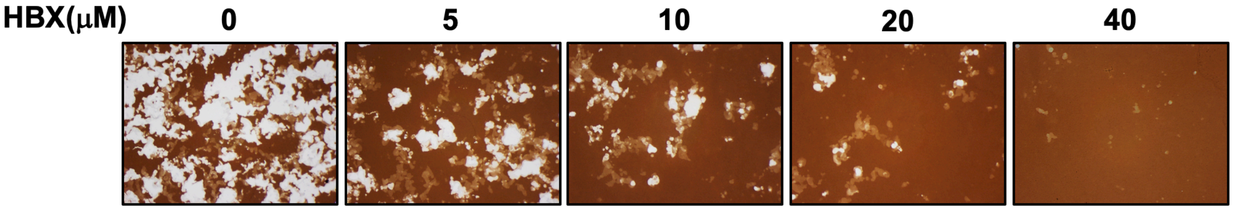


**(B) Osteoclastogenesis**

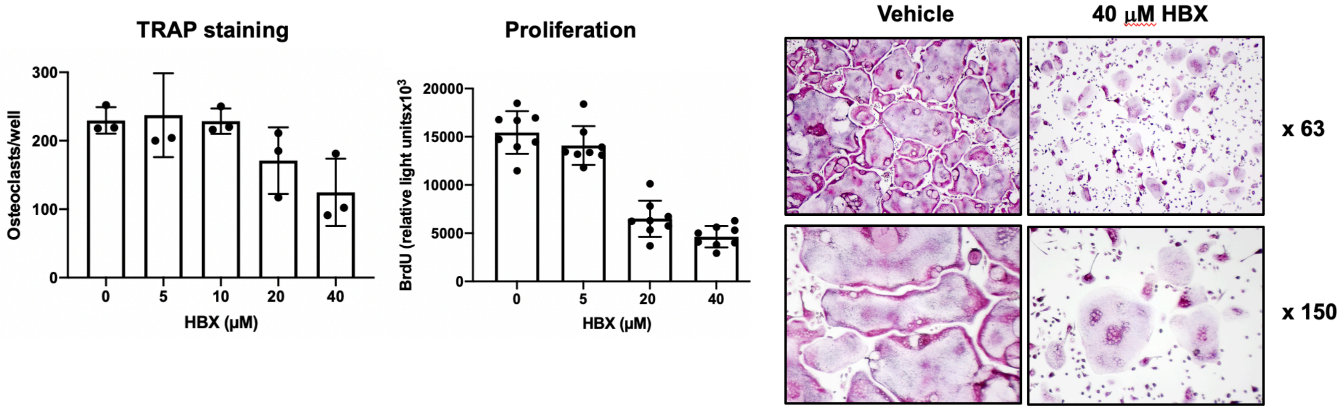

*Supplemental Figure 3.*

***HBX in vitro inhibits osteoblast activity.*** Alizarin red staining of osteoblast activity was quantified against an alizarin red standard. Alkaline phosphatase activity was determined, and proliferation of stromal or calvaria cells was quantified using BrdU (Roche). In each case, HBX inhibited osteoblast proliferation or activity in vitro in a dose dependent fashion.

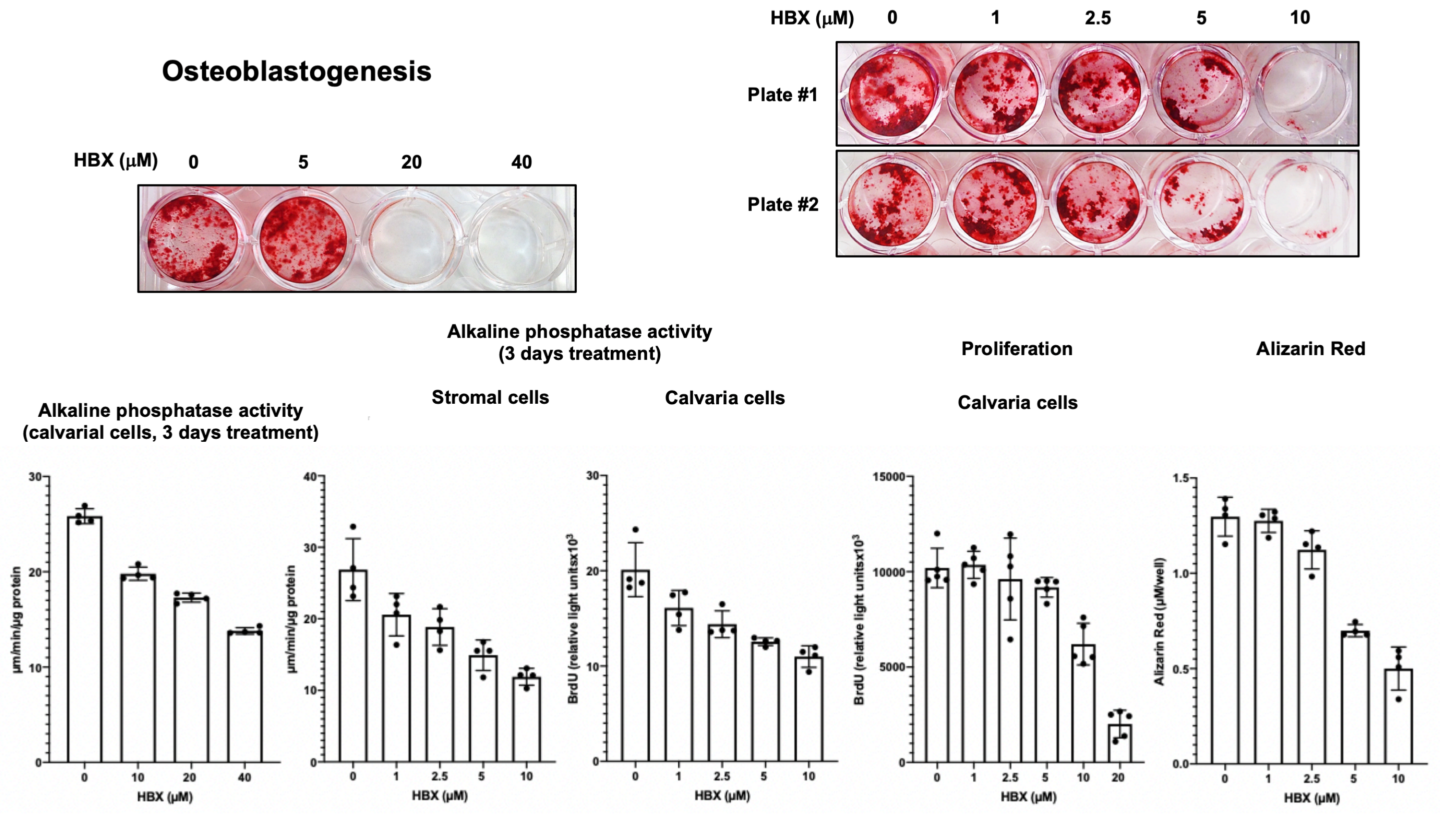

***Supplemental Figure 4.*** Untreated control animals were weighed every 2 weeks from 19 months of age (time 0), through to end of life (~600 days later) to establish rate of change for weight loss. Males - blue, females - red. A fitted line shows a gradual decline in weight from mid-life (19 months of age to late life). 14,958 distinct measures over 677 mice enrolled in the overall study.

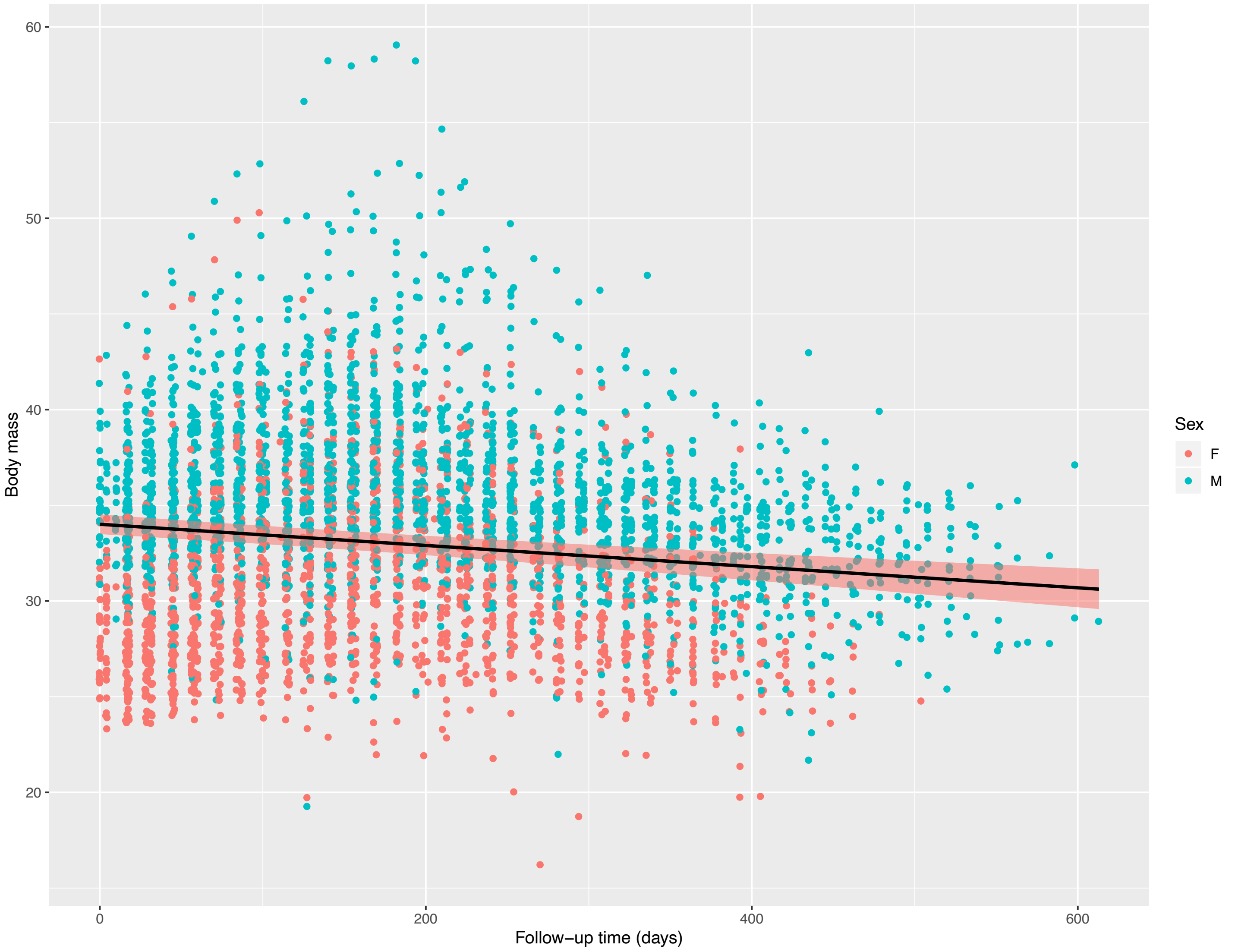

***Supplemental Figure 5.***

Kyphosis changes with age in young versus old, and with age. A) Tortuosity in untreated animals across the lifespan. Numbers under each box plot are the numbers of individual animals measured at each age. B) Plot of an individual animal developing kyphosis over late life showing the sharp increase in tortuosity of the spine. Repeated measures of the animal show relatively little change from 20-30 months of age; however at 30 months of age, tortuosity rapidly changes in concordance with the onset of severe kyphosis.

**A)**

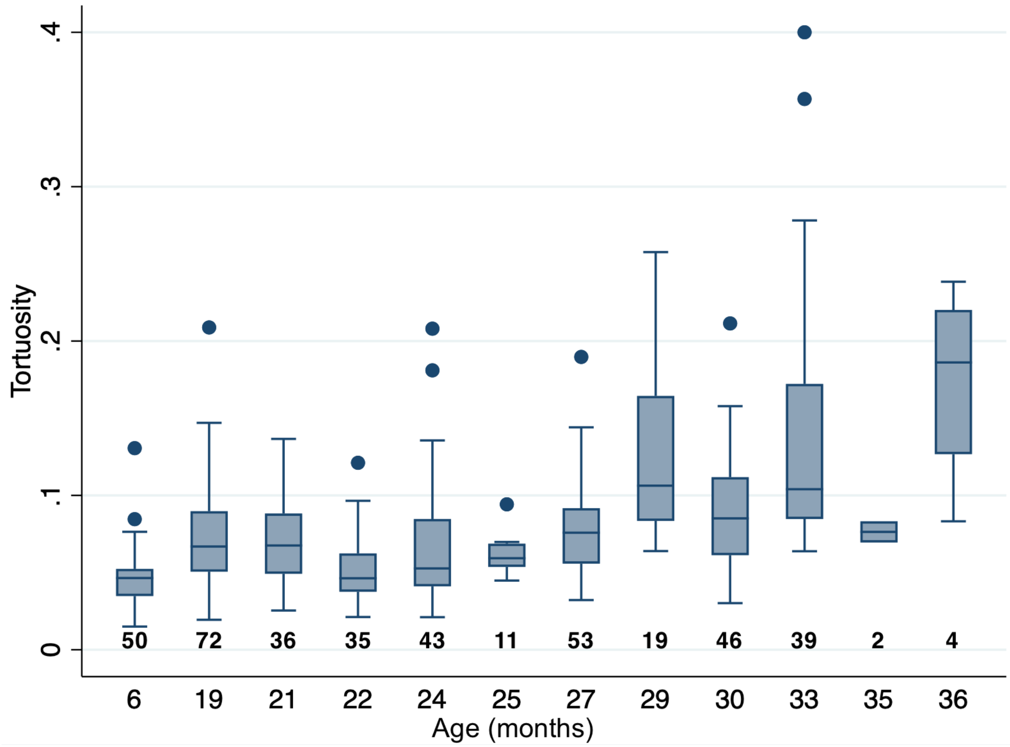


**B)**

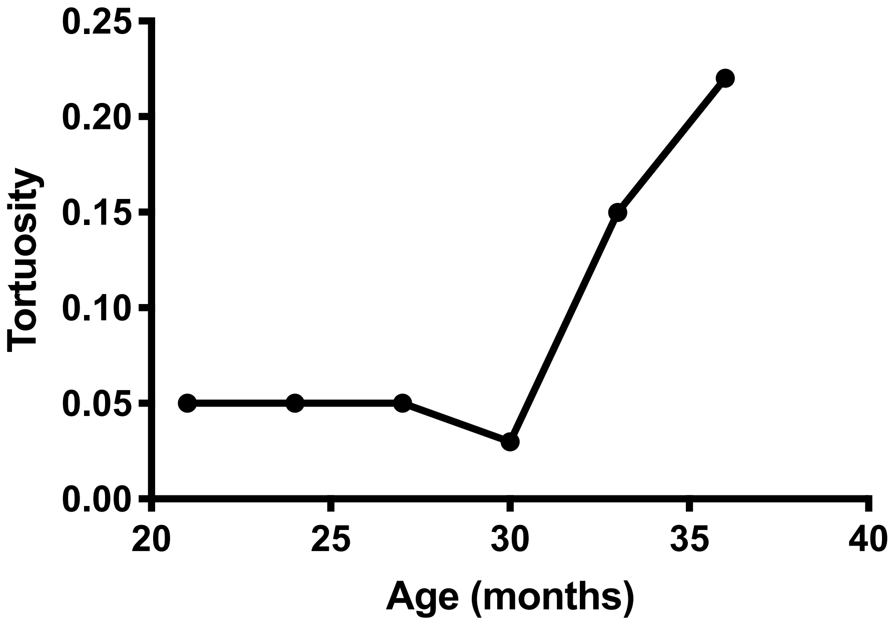

***Supplemental Figure 6. Kyphosis changes in late life.*** A linear model for the rate of change of kyphosis for untreated animals (N=89) was calculated. ß represents the calculated outcome of tortuosity of a 0.01 increase per 100 days on average after adjusting for sex. Sex specific effects were noted (P=0.008), with males (N=48) having a reduced ß in this age range of 0.009 (P=0.0002) versus a ß of 0.02 for females (N=41, P=7.04E-05)

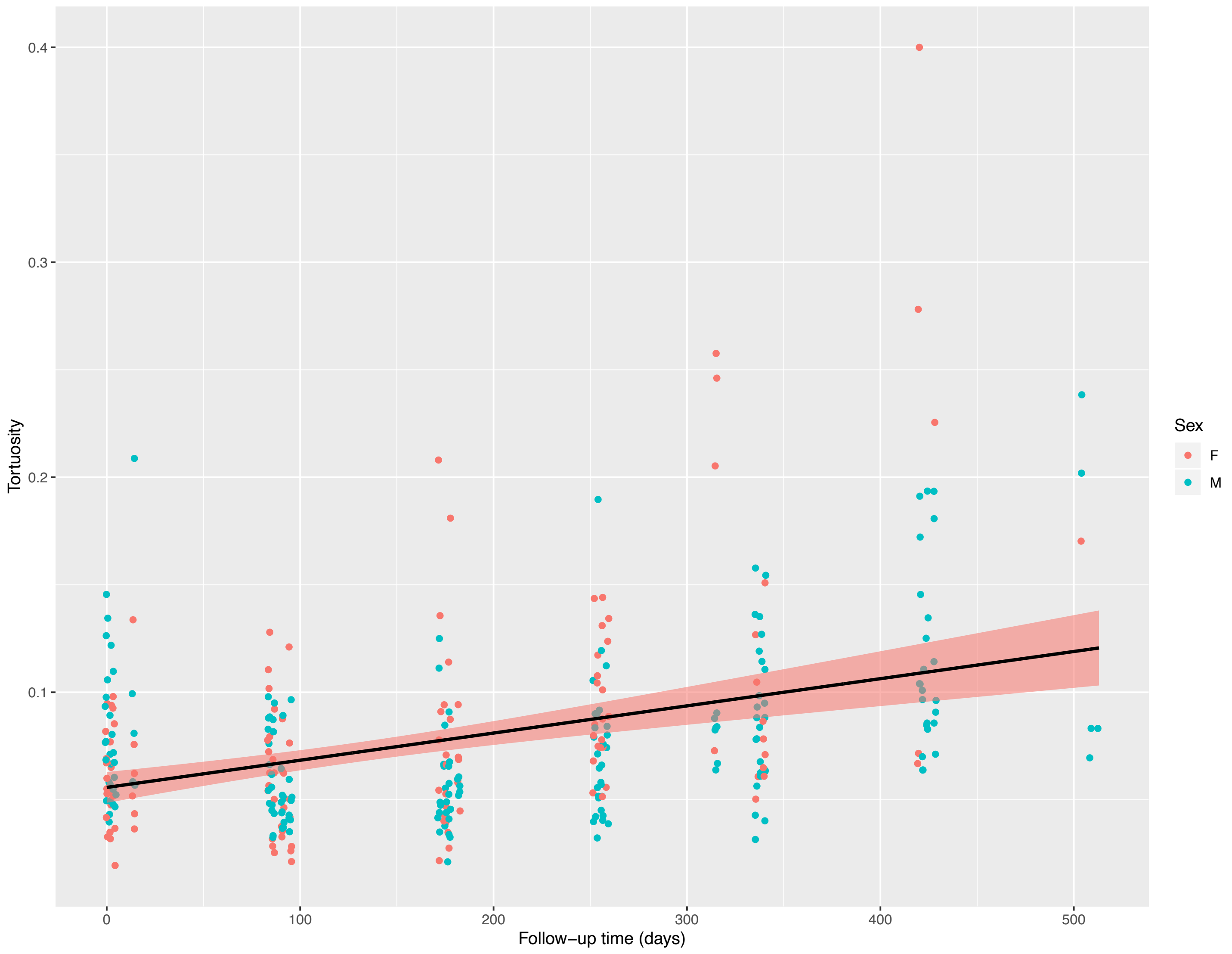

***Supplemental Figure 7. Beta Sito Sterol increases lifespan of D. Melanogaster***. Beta sitosterol (BS), is a plant derived sterol, which when supplemented to Ad libitum (AL) food, increased lifespan in a dose dependent manner in AL fed wild type $w^{1118}$ female flies. YE - yeast extract.

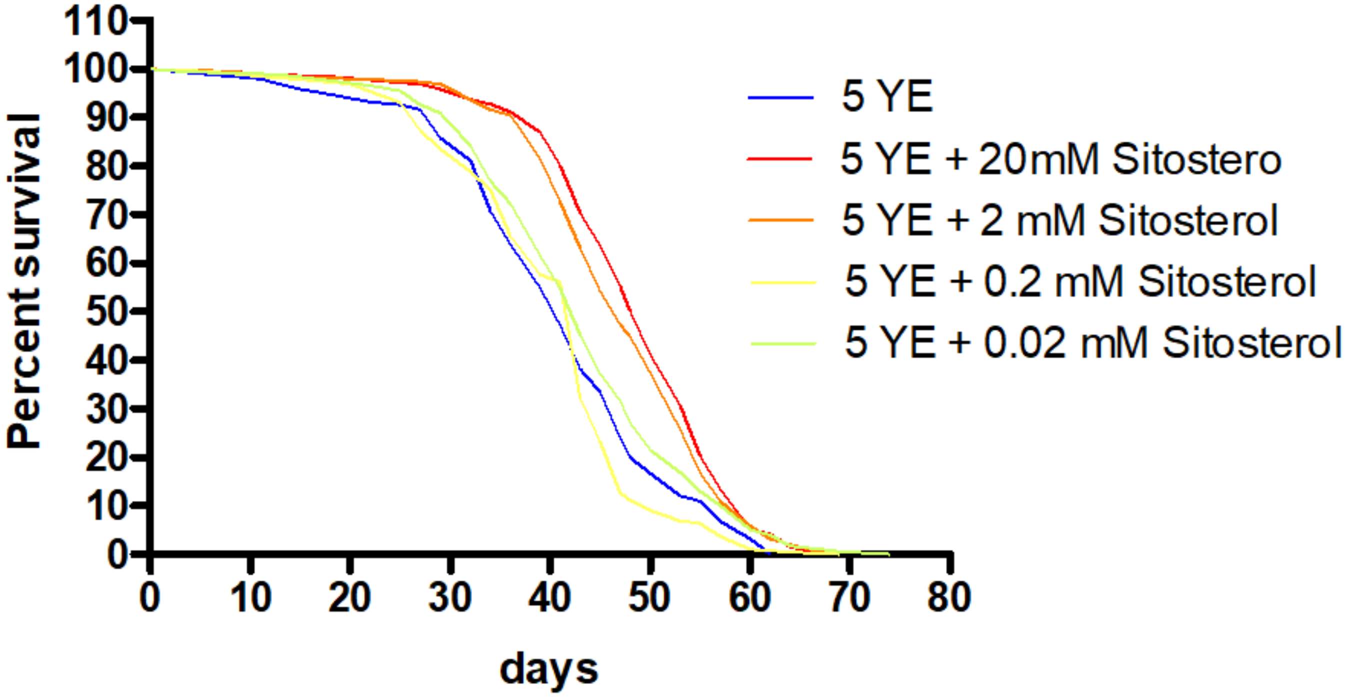

***Supplemental Figure 8***. micro CT of a young femur, showing the region measured in this study. The middle 2mm from the cortical diaphysis was subvolumed from the entire femur, and metrics such as the medullary surface area (A), average cortical thickness (B), cortical surface area (C), D) periosteal circumference (green), or E) endosteal circumference (blue), were calculated.

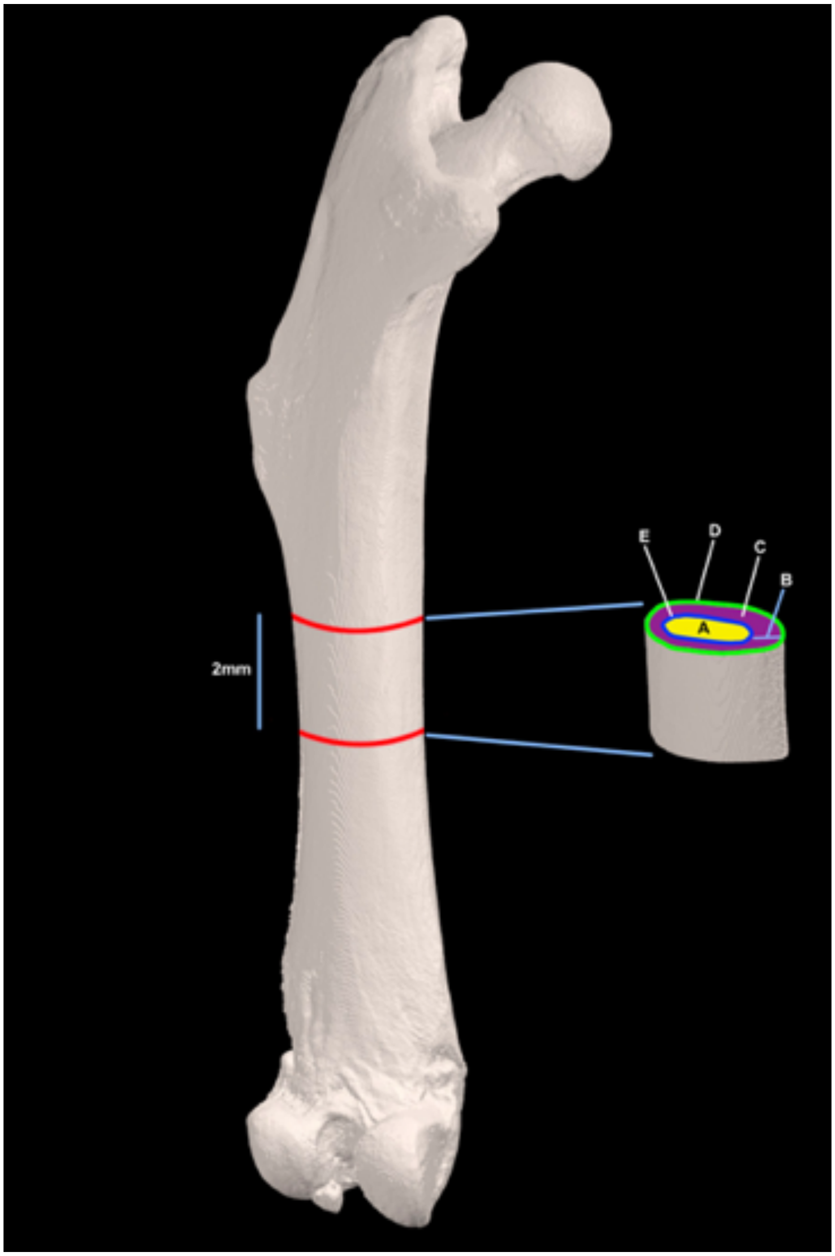

***Supplemental Figure 9.*** A) Lateral, dorsal, posterior views of young and old reconstructed models demonstrating complex changes in 3 dimensions of murine spine showing kyphotic changes relative to a horizontal axis for each view. B) a) lateral view of spine showing L2 and C1 landmarks (red dots) used to construct a 3-D contour of the neural canal tracing 3d changes in spinal contours. b) 3D spline from spine in a). c) Calculated tortuosity of C1-L2 spinal shape in a typical 5 month old mouse, demonstrating a T value of 0.05 for young spine (in 2D).

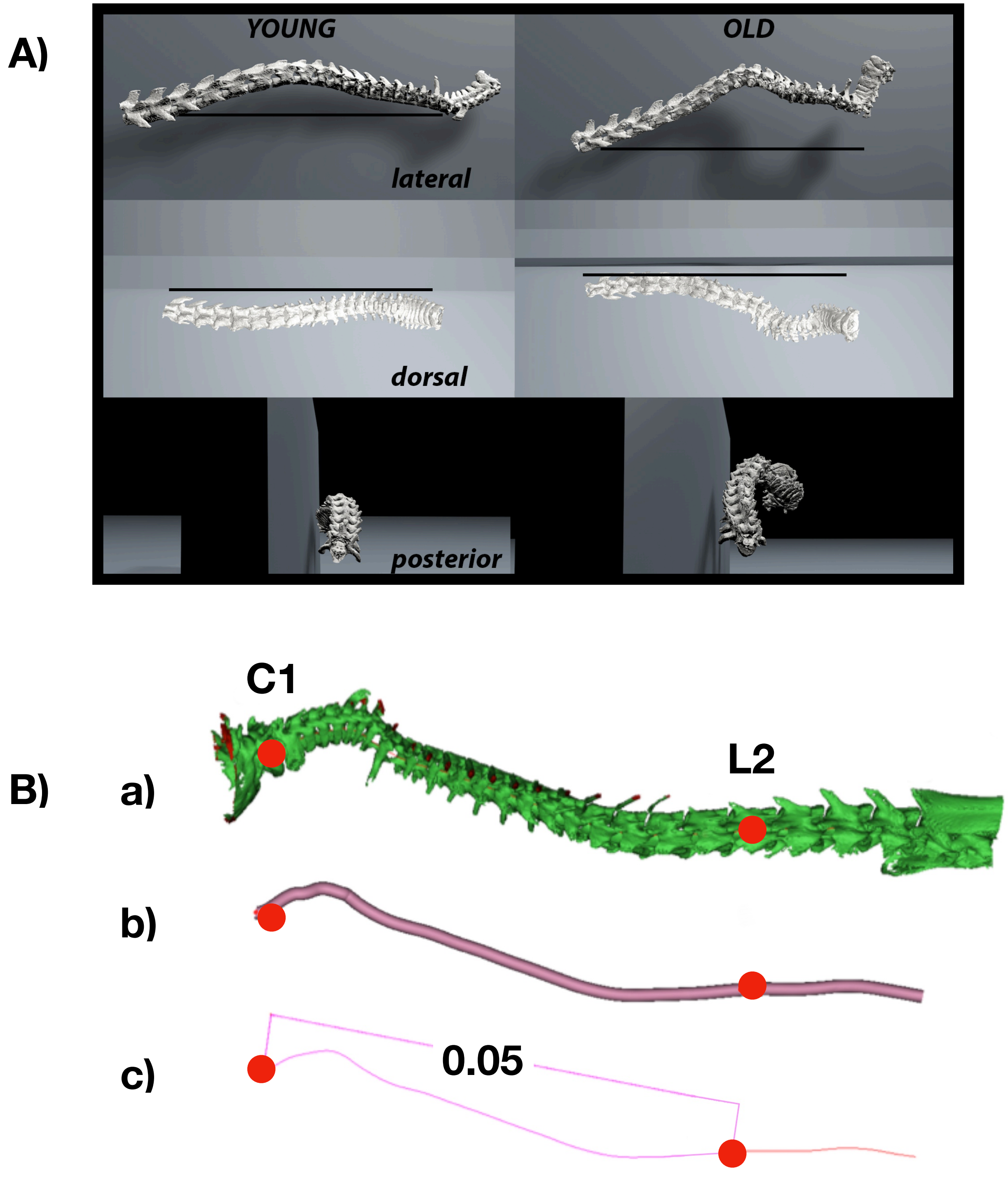

Supplement: Supplementary file 1 — Figure S1 Supporting information [file JBM4-5-e10466-s002.pdf]
