# Supplementary material for: Longitudinal Functional Study of Murine Aging: A Resource for Future Study Designs
Source: JBMR Plus. 2021 Feb 16;5(3):e10466. doi: 10.1002/jbm4.10466 (PMC7990142; doi:10.1002/jbm4.10466)
Supplement: Supplementary file 2 — Table S1 Supporting information [file JBM4-5-e10466-s001.pdf]

Supplemental Tables, Evans et al.

Supplemental Table 1. Variance changes for specific femoral bone traits in 5-month-old mice versus 19-month-old mice

	5m.o. mean	19m.o. mean	P (t-test)	5m.o. variance	19m.o. variance	P (Levene test)
**TV**	4.8	5.7	5.9E-12	0.5	0.2	4.8E-03
**BV**	2.4	2.5	2.7E-03	0.1	0.1	0.4
**BV.TV**	49.5	44.3	8.2E-22	4.8	5.7	0.4
**TS**	18.8	20.7	5.6E-09	3.2	1.4	3.2E-03
**TS.per.**	13.6	14.7	6.0E-09	1.1	0.5	8.4E-04
**BS**	28.4	31.4	2.0E-07	10.1	4.9	2.4E-02
**BS.per.**	23.9	26.5	2.8E-09	5.3	2.6	6.6E-03
**BS.BV**	12.0	12.5	1.0E-03	0.2	1.0	5.9E-03
**T.Ar**	1.9	2.3	5.9E-12	0.1	3.7E-02	4.8E-03
**T.Pm**	5.4	5.9	6.0E-09	0.2	0.1	8.4E-04
**B.Ar**	0.9	1.0	2.7E-03	1.3E-02	1.0E-02	0.4
**B.Pm**	9.6	10.6	2.8E-09	0.8	0.4	6.6E-03
**Ecc**	0.8	0.8	1.5E-18	3.2E-04	8.5E-04	1.4E-03
**Cs.Th**	0.2	0.2	2.8E-03	6.8E-05	2.2E-04	1.7E-03
**Po.cl.**	50.2	55.0	4.1E-14	6.8	10.6	0.4
**endosteal**	4.1	4.7	1.4E-08	0.3	0.2	0.1
**Med.area**	1.0	1.3	1.5E-17	2.7E-02	1.4E-02	1.4E-02

abbreviations: TV - total volume; BV - bone volume; BV.TV - percent bone volume; TS - tissue surface; TS.per. - tissue surface percent; BS - bone surface; BS.per. bone surface percent; BS.BV - bone surface/volume ratio; T.Ar - mean total crossectional tissue area; T.Pm - mean total crossectional tissue perimeter; B.Ar mean total crossectional bone area; B.Pm - mean total crossectional bone perimeter; Ecc - eccentricity; Cs.Th - cross sectional thickness; Po.cl. - closed porosity; endosteal - endosteal perimeter; Med.area - medullary area. N=50 for 5 month old animals, N=64 19 month old animals.

Supplemental table 2. Age-related metrics for mid diaphysis femoral cortical bone in male 19-36 month old untreated animals.

Phenotype	N	ß	SE	P
**TV**	50	0.143	0.008	7.0E-23
**BV**	50	-0.064	0.009	9.7E-09
**BV.TV**	50	-1.838	0.133	3.0E-16
**TS**	50	0.657	0.037	1.2E-21
**TS.per.**	50	0.208	0.009	1.4E-27
**BS**	50	1.426	0.172	8.3E-10
**BS.per.**	50	0.931	0.120	3.9E-09
**BS.BV**	50	0.856	0.068	8.6E-15
**T.Ar**	50	0.057	0.003	6.8E-23
**T.Pm**	50	0.083	0.004	1.4E-27
**B.Ar**	50	-0.026	0.004	9.7E-09
**B.Pm**	50	0.373	0.048	3.9E-09
**Ecc**	50	-0.005	0.001	2.5E-04
**Cs.Th**	50	-0.009	0.001	7.9E-15
**Po.cl.**	50	1.213	0.312	3.5E-04
**endosteal**	50	0.282	0.045	4.1E-07
**Med.area**	50	0.086	0.005	2.1E-20

abbreviations: TV - total volume; BV - bone volume; BV.TV - percent bone volume; TS - tissue surface; TS.per. - tissue surface percent; BS - bone surface; BS.per. bone surface percent; BS.BV - bone surface/volume ratio; T.Ar - mean total crossectional tissue area; T.Pm - mean total crossectional tissue perimeter; B.Ar mean total crossectional bone area; B.Pm - mean total crossectional bone perimeter; Ecc - eccentricity; Cs.Th - cross sectional thickness; Po.cl. - closed porosity; endosteal - endosteal perimeter; Med.area - medullary area; N - total number of male animals measured; ß - calculated mean outcome of phenotype per 100 days between 19 and 36 months of age; SE - standard error of the mean; P - p value

Supplemental table 3. Age-related metrics for mid diaphysis femoral cortical bone in female 19-36 month old untreated animals.

Phenotype	N	ß	SE_females	P
**TV**	39	0.221	0.015	6.5E-17
**BV**	39	-0.033	0.012	0.007
**BV.TV**	39	-2.144	0.174	5.4E-14
**TS**	39	0.800	0.077	7.2E-12
**TS.per.**	39	0.310	0.023	1.6E-15
**BS**	39	1.228	0.302	2.7E-04
**BS.per.**	39	0.874	0.212	2.3E-04
**BS.BV**	39	0.727	0.115	4.1E-07
**T.Ar**	39	0.088	0.006	6.5E-17
**T.Pm**	39	0.124	0.009	1.6E-15
**B.Ar**	39	-0.013	0.005	0.007
**B.Pm**	39	0.350	0.085	2.3E-04
**Ecc**	39	-0.012	0.001	1.5E-09
**Cs.Th**	39	-0.008	0.001	8.7E-08
**Po.cl.**	39	1.550	0.581	0.012
**endosteal**	39	0.223	0.078	0.007
**Med.area**	39	0.102	0.006	6.6E-20

abbreviations: TV - total volume; BV - bone volume; BV.TV - percent bone volume; TS - tissue surface; TS.per. - tissue surface percent; BS - bone surface; BS.per. bone surface percent; BS.BV - bone surface/volume ratio; T.Ar - mean total crossectional tissue area; T.Pm - mean total crossectional tissue perimeter; B.Ar mean total crossectional bone area; B.Pm - mean total crossectional bone perimeter; Ecc - eccentricity; Cs.Th - cross sectional thickness; Po.cl. - closed porosity; endosteal - endosteal perimeter; Med.area - medullary area; N - total number of male animals measured; ß - calculated mean outcome of phenotype per 100 days between 19 and 36 months of age; SE - standard error of the mean; P - p value

Supplemental Tables 4-26; Power calculation of clinically relevant age-related phenotypes.

Power calculation to determine the sample size in each group needed to detect various treatment effects expressed as percent change increase compared to the untreated controls. Power of 0.8 and an alpha level of 0.05 was used, and repeated numbers of measures (N) every 100 days from 19 months of age through 36 months of age to calculate group sizes. Power calculations are based on estimated within and between variation from repeated measures in our control population of aging C57BL/6J mice (max N=138). Variances, and the corresponding power calculations, change for different age-related phenotypes. Supplemental Tables 5-16 (metabolic power tables), show equivalent calculations for multiple metrics over late life. Tables 17-27; femoral bone

Supplemental Table 4. Bone volume percent (BV.TV)

δ	N_9times	N_8times	N_7times	N_6times	N_5times	N_4times	N_3times
0.05	402	406	412	423	446	499	659
0.1	100	101	103	106	112	125	165
0.15	45	45	46	47	50	55	73
0.2	25	25	26	26	28	31	41
0.25	16	16	16	17	18	20	26
0.3	11	11	11	12	12	14	18
0.35	8	8	8	9	9	10	13
0.4	6	6	6	7	7	8	10

Supplemental Table 5. Blood glucose (BG)

δ	N_9times	N_8times	N_7times	N_6times	N_5times	N_4times	N_3times
0.05	644	666	702	768	900	1208	2131
0.1	161	166	176	192	225	302	533
0.15	72	74	78	85	100	134	237
0.2	40	42	44	48	56	75	133
0.25	26	27	28	31	36	48	85
0.3	18	18	20	21	25	34	59
0.35	13	14	14	16	18	25	43
0.4	10	10	11	12	14	19	33

power tables. If more than ~100 animals per group were calculated to detect significant

Supplemental Table 6. EE.BMN (energy expenditure/body mass at night)

δ	N_9times	N_8times	N_7times	N_6times	N_5times	N_4times	N_3times
**0.05**	2820	2850	2900	2990	3169	3588	4845
**0.1**	705	713	725	747	792	897	1211
**0.15**	313	317	322	332	352	399	538
**0.2**	176	178	181	187	198	224	303
**0.25**	113	114	116	120	127	144	194
**0.3**	78	79	81	83	88	100	135
**0.35**	58	58	59	61	65	73	99
**0.4**	44	45	45	47	50	56	76

Supplemental Table 7. EE.LBMN (energy expenditure/lean body mass night)

δ	N_9times	N_8times	N_7times	N_6times	N_5times	N_4times	N_3times
**0.05**	4110	4141	4192	4284	4469	4898	6187
**0.1**	1028	1035	1048	1071	1117	1225	1547
**0.15**	457	460	466	476	497	544	687
**0.2**	257	259	262	268	279	306	387
**0.25**	164	166	168	171	179	196	247
**0.3**	114	115	116	119	124	136	172
**0.35**	84	85	86	87	91	100	126
**0.4**	64	65	66	67	70	77	97

changes, these variables are not shown. Red box, main text example.

Supplemental table 8. EE (energy expenditure by night)

δ	N_9times	N_8times	N_7times	N_6times	N_5times	N_4times	N_3times
**0.05**	1348	1381	1436	1535	1734	2196	3583
**0.1**	337	345	359	384	433	549	896
**0.15**	150	153	160	171	193	244	398
**0.2**	84	86	90	96	108	137	224
**0.25**	54	55	57	61	69	88	143
**0.3**	37	38	40	43	48	61	100
**0.35**	28	28	29	31	35	45	73
**0.4**	21	22	22	24	27	34	56

Supplemental Table 9. PMN (meters walked at night)

δ	N_9times	N_8times	N_7times	N_6times	N_5times	N_4times	N_3times
**0.05**	259	312	400	559	878	1620	3848
**0.1**	65	78	100	140	219	405	962
**0.15**	29	35	44	62	98	180	428
**0.2**	16	19	25	35	55	101	240
**0.25**	10	12	16	22	35	65	154
**0.3**	7	9	11	16	24	45	107
**0.35**	5	6	8	11	18	33	79
**0.4**	4	5	6	9	14	25	60

Supplemental Table 10. PSD (speed of walking in day)

δ	N_9times	N_8times	N_7times	N_6times	N_5times	N_4times	N_3times
0.05	626	652	696	775	933	1301	2407
0.1	156	163	174	194	233	325	602
0.15	70	72	77	86	104	145	267
0.2	39	41	43	48	58	81	150
0.25	25	26	28	31	37	52	96
0.3	17	18	19	22	26	36	67
0.35	13	13	14	16	19	27	49
0.4	10	10	11	12	15	20	38

Supplemental Table 11. PSN (speed of walking at night)

δ	N_9times	N_8times	N_7times	N_6times	N_5times	N_4times	N_3times
0.05	662	702	768	887	1125	1680	3345
0.1	166	175	192	222	281	420	836
0.15	74	78	85	99	125	187	372
0.2	41	44	48	55	70	105	209
0.25	26	28	31	35	45	67	134
0.3	18	20	21	25	31	47	93
0.35	14	14	16	18	23	34	68
0.4	10	11	12	14	18	26	52

Supplemental Table 12. RQN (respiratory quotient at night)

δ	N_9times	N_8times	N_7times	N_6times	N_5times	N_4times	N_3times
0.05	717	748	799	892	1078	1512	2813
0.1	179	187	200	223	270	378	703
0.15	80	83	89	99	120	168	313
0.2	45	47	50	56	67	94	176
0.25	29	30	32	36	43	60	113
0.3	20	21	22	25	30	42	78
0.35	15	15	16	18	22	31	57
0.4	11	12	12	14	17	24	44

Supplemental Table 13. VO2N (VO2 at night)

δ	N_9times	N_8times	N_7times	N_6times	N_5times	N_4times	N_3times
0.05	587.17	613.92	658.51	738.77	899.28	1273.81	2397.39
0.1	146.79	153.48	164.63	184.69	224.82	318.45	599.35
0.15	65.24	68.21	73.17	82.09	99.92	141.53	266.38
0.2	36.7	38.37	41.16	46.17	56.2	79.61	149.84
0.25	23.49	24.56	26.34	29.55	35.97	50.95	95.9
0.3	16.31	17.05	18.29	20.52	24.98	35.38	66.59
0.35	11.98	12.53	13.44	15.08	18.35	26	48.93
0.4	9.17	9.59	10.29	11.54	14.05	19.9	37.46

Supplemental Table 14. WMN (wheel meters run at night)

δ	N_9times	N_8times	N_7times	N_6times	N_5times	N_4times	N_3times
**0.05**	1102	1118	1144	1192	1287	1509	2176
**0.1**	275	279	286	298	322	377	544
**0.15**	122	124	127	132	143	168	242
**0.2**	69	70	72	74	80	94	136
**0.25**	44	45	46	48	51	60	87
**0.3**	31	31	32	33	36	42	60
**0.35**	22	23	23	24	26	31	44
**0.4**	17	17	18	19	20	24	34

Supplemental Table 15. WSN (wheel speed run at night)

δ	N_9times	N_8times	N_7times	N_6times	N_5times	N_4times	N_3times
**0.05**	1312	1324	1342	1376	1443	1600	2071
**0.1**	328	331	336	344	361	400	518
**0.15**	146	147	149	153	160	178	230
**0.2**	82	83	84	86	90	100	129
**0.25**	52	53	54	55	58	64	83
**0.3**	36	37	37	38	40	44	58
**0.35**	27	27	27	28	29	33	42
**0.4**	21	21	21	22	23	25	32

Supplemental Table 16. BV (bone volume)

δ	N_9times	N_8times	N_7times	N_6times	N_5times	N_4times	N_3times
**0.05**	1445	1468	1507	1577	1717	2044	3024
**0.1**	361	367	377	394	429	511	756
**0.15**	161	163	167	175	191	227	336
**0.2**	90	92	94	99	107	128	189
**0.25**	58	59	60	63	69	82	121
**0.3**	40	41	42	44	48	57	84
**0.35**	29	30	31	32	35	42	62
**0.4**	23	23	24	25	27	32	47

Supplemental Table 17. TV (total volume)

δ	N_9times	N_8times	N_7times	N_6times	N_5times	N_4times	N_3times
**0.05**	566	567	570	575	585	608	678
**0.1**	141	142	143	144	146	152	170
**0.15**	63	63	63	64	65	68	75
**0.2**	35	35	36	36	37	38	42
**0.25**	23	23	23	23	23	24	27
**0.3**	16	16	16	16	16	17	19
**0.35**	12	12	12	12	12	12	14
**0.4**	9	9	9	9	9	10	11

Supplemental Table 18. BV.TV (Bone volume/total volume)

δ	N_9times	N_8times	N_7times	N_6times	N_5times	N_4times	N_3times
0.05	402	406	412	423	446	499	659
0.1	100	101	103	106	112	125	165
0.15	45	45	46	47	50	55	73
0.2	25	25	26	26	28	31	41
0.25	16	16	16	17	18	20	26
0.3	11	11	11	12	12	14	18
0.35	8	8	8	9	9	10	13
0.4	6	6	6	7	7	8	10

Supplemental Table 19. TS (Tissue surface)

δ	N_9times	N_8times	N_7times	N_6times	N_5times	N_4times	N_3times
0.05	61	67	75	90	120	191	403
0.1	15	17	19	23	30	48	101
0.15	7	7	8	10	13	21	45
0.2	4	4	5	6	8	12	25
0.25	2	3	3	4	5	8	16
0.3	2	2	2	3	3	5	11
0.35	1	1	2	2	2	4	8
0.4	1	1	1	1	2	3	6

Supplemental Table 20. TS.per. (Tissue percent)

δ	N_9times	N_8times	N_7times	N_6times	N_5times	N_4times	N_3times
**0.05**	244	246	250	258	273	307	410
**0.1**	61	62	63	64	68	77	103
**0.15**	27	27	28	29	30	34	46
**0.2**	15	15	16	16	17	19	26
**0.25**	10	10	10	10	11	12	16
**0.3**	7	7	7	7	8	9	11
**0.35**	5	5	5	5	6	6	8
**0.4**	4	4	4	4	4	5	6

Supplemental Table 21. BS.BV (Bone surface/Bone volume)

δ	N_9times	N_8times	N_7times	N_6times	N_5times	N_4times	N_3times
**0.05**	1056	1064	1076	1097	1140	1240	1541
**0.1**	264	266	269	274	285	310	385
**0.15**	117	118	120	122	127	138	171
**0.2**	66	66	67	69	71	78	96
**0.25**	42	43	43	44	46	50	62
**0.3**	29	30	30	30	32	34	43
**0.35**	22	22	22	22	23	25	31
**0.4**	17	17	17	17	18	19	24

Supplemental Table 22. TA (Tissue area)

δ	N_9times	N_8times	N_7times	N_6times	N_5times	N_4times	N_3times
0.05	566	567	570	575	585	608	678
0.1	141	142	143	144	146	152	170
0.15	63	63	63	64	65	68	75
0.2	35	35	36	36	37	38	42
0.25	23	23	23	23	23	24	27
0.3	16	16	16	16	16	17	19
0.35	12	12	12	12	12	12	14
0.4	9	9	9	9	9	10	11

Supplemental Table 23. T.Pm (Tissue perimeter)

δ	N_9times	N_8times	N_7times	N_6times	N_5times	N_4times	N_3times
0.05	244	247	251	258	273	307	411
0.1	61	62	63	65	68	77	103
0.15	27	27	28	29	30	34	46
0.2	15	15	16	16	17	19	26
0.25	10	10	10	10	11	12	16
0.3	7	7	7	7	8	9	11
0.35	5	5	5	5	6	6	8
0.4	4	4	4	4	4	5	6

Supplemental Table 24. Ecc. (Eccentricity)

δ	N_9times	N_8times	N_7times	N_6times	N_5times	N_4times	N_3times
**0.05**	350	396	472	609	882	1520	3433
**0.1**	88	99	118	152	220	380	858
**0.15**	39	44	52	68	98	169	381
**0.2**	22	25	29	38	55	95	215
**0.25**	14	16	19	24	35	61	137
**0.3**	10	11	13	17	25	42	95
**0.35**	7	8	10	12	18	31	70
**0.4**	5	6	7	10	14	24	54

Supplemental Table 25. Cs.Th (cross sectional thickness)

δ	N_9times	N_8times	N_7times	N_6times	N_5times	N_4times	N_3times
**0.05**	812	819	831	852	893	990	1280
**0.1**	203	205	208	213	223	247	320
**0.15**	90	91	92	95	99	110	142
**0.2**	51	51	52	53	56	62	80
**0.25**	33	33	33	34	36	40	51
**0.3**	23	23	23	24	25	27	36
**0.35**	17	17	17	17	18	20	26
**0.4**	13	13	13	13	14	15	20

Supplemental Table 26. Med.area (medullary area)

δ	N_9times	N_8times	N_7times	N_6times	N_5times	N_4times	N_3times
**0.05**	494	495	497	501	508	526	577
**0.1**	124	124	124	125	127	131	144
**0.15**	55	55	55	56	57	58	64
**0.2**	31	31	31	31	32	33	36
**0.25**	20	20	20	20	20	21	23
**0.3**	14	14	14	14	14	15	16
**0.35**	10	10	10	10	10	11	12
**0.4**	8	8	8	8	8	8	9

Supplemental Table 27. Metabolic measures assayed

Phenotype	Units	Description
BG	mg/dl	Blood glucose levels (fasted)
BMD	g	Body mass by day (average)
BMN	g	Body mass by night (average)
EE.BMD	kcal/hr/g	Energy expenditure by day/body mass (average)
EE.BMN	kcal/hr/g	Energy expenditure/body mass by night (average)
EE.LBMD	kcal/hr/g	Energy expenditure/lean body mass by day (average)
EE.LBMN	kcal/hr/g	Energy expenditure/lean body mass by night (average)
EED	kcal/hr	Energy expenditure by day (average)
EEN	kcal/hr	Energy expenditure by night (average)
FBM	g	Fat mass (estimated by QMR)
FD	g	Food consumed by day (average)
FN	g	Food consumed by night (average)
LBM	g	Lean body mass
PMD	m	Meters walked by day (average)
PMN	m	Meters walked by night (average)
PSD	m/s	Mean speed of walking by day (average)
PSN	m/s	Mean speed of walking by night (average)
RQD	Dimensionless number	Respiratory quotient by day (average)
RQN	Dimensionless number	Respiratory quotient by night (average)
SD	hours(h)	Sleep by day (average)
SN	hours(h)	Sleep by night (average)
VCD	ml/min	VCO_2 by day (average)
VCN	ml/min	VCO_2 by night (average)
VOD	ml/min	VO_2 by day (average)
VON	ml/min	VO_2 by night (average)
WD	g	Water drunk by day (average)
WN	g	Water drunk by night (average)
WMD	m	Meters run in a wheel by day (average)
WMN	m	Meters run in a wheel by night (average)
WSD	m/s	Speed of running in a wheel by day (average)
WSN	m/s	Speed of running a wheel by night (average)

Supplemental Table 28. Metabolic changes with age; males

Phenotype	Total N	ß	SE	P	N (5 months old)	N (19 months old)
**BG**	52	5.340	9.516	0.577	16	36
**EE.BMD**	28	-0.001	4.0E-04	0.085	16	12
**EE.BMN**	28	-0.003	0.001	0.001	16	12
**EE.LBMD**	28	-3.2E-04	4.5E-04	0.478	16	12
**EE.LBMN**	28	-0.003	0.001	0.003	16	12
**EED**	28	0.024	0.018	0.185	16	12
**EEN**	28	-0.032	0.026	0.217	16	12
**FBM**	52	2.419	0.615	2.6E-04	16	36
**FD**	28	-0.174	0.172	0.323	16	12
**FN**	28	0.321	0.262	0.232	16	12
**LBM**	52	1.238	0.546	0.028	16	36
**PMD**	28	6.720	4.936	0.185	16	12
**PMN**	28	-4.966	8.420	0.560	16	12
**PSD**	28	-0.001	0.001	0.427	16	12
**PSN**	28	-0.001	0.001	0.125	16	12
**RQD**	28	-0.051	0.024	0.044	16	12
**RQN**	28	-0.053	0.018	0.007	16	12
**SD**	28	-0.432	0.706	0.546	16	12
**SN**	28	-0.144	0.272	0.601	16	12
**VCD**	28	-0.005	0.070	0.938	16	12
**VCN**	28	-0.203	0.096	0.044	16	12
**VOD**	28	0.102	0.057	0.084	16	12
**VON**	28	-0.080	0.083	0.347	16	12
**WD**	28	0.091	0.117	0.444	16	12
**WMD**	27	-160	97	0.112	15	12
**WMN**	27	-1080	530	0.052	15	12
**WN**	28	-0.325	0.232	0.174	16	12
**WSD**	26	-0.011	0.016	0.521	15	11
**WSN**	26	-0.025	0.014	0.083	15	11

abbreviations: Total N; Total numbers of animals per measured phenotype are shown; ß - change relative to young animals; SE - standard error; P - P value; (*Phenotype*)D - average by day; (*Phenotype*)N - average by night; BG - fasted blood glucose level; BM - body mass; EE.BM - energy expenditure/body mass; EE.LBM - energy expenditure/ lean body mass; EE - energy expenditure; FBM - fat; F - food consumed; LBM - lean body mass; PM - meters walked; PS - mean speed of walking; RQ - respiratory quotient; S - sleep; VC - VCO2; VO - VO2; W - water drunk; WM - wheel meters run; WS - wheel speed while running.

Supplemental Table 29. Metabolic changes with age; females

Phenotype	Total N	ß	SE	P	N (5 months old)	N (19 months old)
**BG**	45	0.7	8.4	0.938	16	29
**EE.BMD**	28	-0.001	0.001	0.188	16	12
**EE.BMN**	28	-0.006	0.001	4.9E-05	16	12
**EE.LBMD**	28	-0.001	0.001	0.240	16	12
**EE.LBMN**	28	-0.006	0.001	1.4E-04	16	12
**EED**	28	0.025	0.016	0.128	16	12
**EEN**	28	-0.068	0.032	0.046	16	12
**FBM**	45	2.1	0.4	7.3E-06	16	29
**FD**	28	-0.483	0.238	0.053	16	12
**FN**	28	-0.769	0.337	0.031	16	12
**LBM**	45	1.8	0.3	1.3E-07	16	29
**PMD**	28	-4.9	2.6	0.075	16	12
**PMN**	28	-5.5	13.4	0.685	16	12
**PSD**	28	-0.001	0.001	0.134	16	12
**PSN**	28	-0.001	0.001	0.387	16	12
**RQD**	28	-0.234	0.023	1.3E-10	16	12
**RQN**	28	-0.232	0.027	4.5E-09	16	12
**SD**	28	0.618	0.480	0.209	16	12
**SN**	28	1.548	0.364	2.4E-04	16	12
**VCD**	28	-0.194	0.063	0.005	16	12
**VCN**	28	-0.586	0.129	1.1E-04	16	12
**VOD**	28	0.158	0.050	0.004	16	12
**VON**	28	-0.122	0.102	0.246	16	12
**WD**	28	0.019	0.169	0.913	16	12
**WMD**	26	-121	123	0.332	14	12
**WMN**	26	-5448	740	1.3E-07	14	12
**WN**	28	-0.217	0.177	0.231	16	12
**WSD**	26	-0.056	0.023	0.021	14	12
**WSN**	26	-0.102	0.015	2.7E-07	14	12

abbreviations: Total N; Total numbers of animals per measured phenotype are shown; ß - change relative to young animals; SE - standard error; P - P value; (*Phenotype*)D - average by day; (*Phenotype*)N - average by night; BG - fasted blood glucose level; BM - body mass; EE.BM - energy expenditure/body mass; EE.LBM - energy expenditure/ lean body mass; EE - energy expenditure; FBM - fat; F - food consumed; LBM - lean body mass; PM - meters walked; PS - mean speed of walking; RQ - respiratory quotient; S - sleep; VC - VCO2; VO - VO2; W - water drunk; WM - wheel meters run; WS - wheel speed while running.

Supp. Table 30. Metabolic changes with advanced age

Phenotype	N	ß	SE	P	P (Sex Interaction)
**BG**	69	-12	1.461	7.1E-12	0.477
**EE.BMD**	37	5.8E-05	1.2E-04	0.628	0.004
**EE.BMN**	37	-0.001	1.7E-04	0.001	0.527
**EE.LBMD**	36	0.000	2.1E-04	0.971	0.000
**EE.LBMN**	36	-0.002	4.3E-04	0.001	0.003
**EED**	40	0.004	0.005	0.395	6.1E-08
**EEN**	40	-0.020	0.005	2.4E-04	8.3E-05
**FBM**	69	-0.610	0.121	2.7E-06	0.075
**FD**	41	0.053	0.037	0.160	0.429
**FN**	41	0.002	0.081	0.982	0.898
**LBM**	69	0.077	0.093	0.411	0.440
**PMD**	41	-4.205	0.987	1.4E-04	0.012
**PMN**	41	-7.342	1.870	3.5E-04	0.231
**PSD**	41	-0.001	1.3E-04	1.6E-05	0.079
**PSN**	41	-0.001	1.7E-04	1.1E-04	0.247
**RQD**	40	0.013	0.004	0.004	0.633
**RQN**	40	0.017	0.004	1.9E-05	0.782
**SD**	41	-0.106	0.160	0.512	0.002
**SN**	41	0.184	0.124	0.147	0.109
**VCD**	40	0.022	0.016	0.171	5.5E-07
**VCN**	40	-0.035	0.016	0.040	0.001
**VOD**	40	0.012	0.018	0.495	8.6E-08
**VON**	40	-0.075	0.016	3.9E-05	6.9E-05
**WD**	41	0.105	0.047	0.032	0.094
**WMD**	40	-14	6.446	0.036	0.404
**WMN**	40	-604	100.906	3.7E-07	0.365
**WN**	41	0.152	0.102	0.146	0.024
**WSD**	36	-0.011	0.004	0.004	0.512
**WSN**	37	-0.019	0.003	5.1E-07	0.947

abbreviations: Total N; Total numbers of animals per measured phenotype are shown; ß - mean change in outcome over 100 days; SE - standard error; P - P value; P(Sex interaction) - P <0.05 ß significantly different by sex; (*Phenotype*)D - average by day; (*Phenotype*)N - average by night; BG - fasted blood glucose level; BM - body mass; EE.BM - energy expenditure/body mass; EE.LBM - energy expenditure/lean body mass; EE - energy expenditure; FBM - fat; F - food consumed; LBM - lean body mass; PM - meters walked; PS - mean speed of walking; RQ - respiratory quotient; S - sleep; VC - VCO2; VO - VO2; W - water drunk; WM - wheel meters run; WS - wheel speed while running.

Supplemental Table 31. Metabolic changes in males of advanced age

Phenotype	N	ß	SE	P
**BG**	41	-11.2	1.7	1.1E-07
**EE.BMD**	18	-2.2E-04	1.1E-04	0.079
**EE.BMN**	18	-0.001	1.5E-04	2.8E-04
**EE.LBMD**	18	-0.001	2.2E-04	0.009
**EE.LBMN**	18	-0.003	4.9E-04	1.3E-04
**EED**	21	-0.014	0.004	0.003
**EEN**	21	-0.032	0.005	1.0E-05
**FBM**	41	-0.755	0.129	1.2E-06
**FD**	21	0.030	0.042	0.496
**FN**	21	-0.015	0.065	0.823
**LBM**	41	0.029	0.118	0.808
**PMD**	21	-6.1	1.4	0.001
**PMN**	21	-5.9	2.2	0.018
**PSD**	21	-4.7E-04	1.6E-04	0.011
**PSN**	21	-5.9E-04	2.2E-04	0.020
**RQD**	21	0.010	0.005	0.094
**RQN**	21	0.017	0.004	0.001
**SD**	21	0.262	0.198	0.203
**SN**	21	0.349	0.168	0.058
**VCD**	21	-0.032	0.014	0.038
**VCN**	21	-0.072	0.017	0.001
**VOD**	21	-0.051	0.013	0.002
**VON**	21	-0.117	0.017	4.2E-06
**WD**	21	0.046	0.042	0.295
**WMD**	20	-18	9	0.056
**WMN**	20	-515	117	4.9E-04
**WN**	21	-0.018	0.103	0.865
**WSD**	17	-0.013	0.004	0.014
**WSN**	19	-0.019	0.003	0.000

abbreviations: Total N; Total numbers of animals per measured phenotype are shown; ß - mean change in outcome over 100 days; SE - standard error; P - P value; (*Phenotype*)D - average by day; (*Phenotype*)N - average by night; BG - fasted blood glucose level; BM - body mass; EE.BM - energy expenditure/body mass; EE.LBM - energy expenditure/ lean body mass; EE - energy expenditure; FBM - fat; F - food consumed; LBM - lean body mass; PM - meters walked; PS - mean speed of walking; RQ - respiratory quotient; S - sleep; VC - VCO2; VO - VO2; W - water drunk; WM - wheel meters run; WS - wheel speed while running.

Supplemental Table 32. Metabolic changes in females of advanced age

Phenotype	N	ß	SE	P
**BG**	28	-14	3	2.8E-04
**EE.BMD**	19	0.000	2.2E-04	0.054
**EE.BMN**	19	-0.000	3.5E-04	0.204
**EE.LBMD**	18	0.001	3.8E-04	0.005
**EE.LBMN**	18	-0.000	0.001	0.899
**EED**	19	0.032	0.008	0.001
**EEN**	19	0.006	0.009	0.533
**FBM**	28	-0.304	0.263	0.257
**FD**	20	0.091	0.070	0.216
**FN**	20	0.008	0.186	0.965
**LBM**	28	0.225	0.152	0.152
**PMD**	20	-0.933	0.881	0.308
**PMN**	20	-10	3	0.012
**PSD**	20	-0.001	2.3E-04	0.001
**PSN**	20	-0.001	2.4E-04	0.001
**RQD**	19	0.015	0.006	0.038
**RQN**	19	0.016	0.007	0.046
**SD**	20	-0.735	0.204	0.003
**SN**	20	-0.057	0.176	0.752
**VCD**	19	0.100	0.021	3.3E-04
**VCN**	19	0.047	0.033	0.167
**VOD**	19	0.108	0.026	0.001
**VON**	19	0.013	0.030	0.674
**WD**	20	0.206	0.107	0.075
**WMD**	20	-10	9	0.299
**WMN**	20	-813	234	0.003
**WN**	20	0.440	0.190	0.037
**WSD**	19	-0.012	0.007	0.102
**WSN**	18	-0.018	0.006	0.008

abbreviations: Total N; Total numbers of animals per measured phenotype are shown; ß - mean change in outcome over 100 days; SE - standard error; P - P value; (*Phenotype*)D - average by day; (*Phenotype*)N - average by night; BG - fasted blood glucose level; BM - body mass; EE.BM - energy expenditure/body mass; EE.LBM - energy expenditure/ lean body mass; EE - energy expenditure; FBM - fat; F - food consumed; LBM - lean body mass; PM - meters walked; PS - mean speed of walking; RQ - respiratory quotient; S - sleep; VC - VCO2; VO - VO2; W - water drunk; WM - wheel meters run; WS - wheel speed while running.

Supplemental Table 33. Statistically significant metabolic changes with late life

Phenotype	Intervention	N Control	N Intervention	ß	SE	P	P (sex interaction)
**BG**	Clioquinol	69	17	13	4	0.001	0.075
**EE.BMD**	Lithium	37	7	0.001	0.000	0.042	0.007
**FD**	HBX	41	10	-0.216	0.096	0.029	0.040
**PSN**	HBX	41	10	-0.001	4.7E-04	0.017	0.169
**RQD**	Lithium	40	7	-0.054	0.010	3.2E-06	0.722
**RQD**	HBX	40	10	-0.021	0.009	0.024	0.126
**RQN**	Lithium	40	9	-0.044	0.010	1.1E-04	0.488
**RQN**	HBX	40	10	-0.021	0.008	0.015	0.756
**SD**	Beta sitosterol	41	20	-0.605	0.256	0.022	1.4E-07
**SD**	HBX	41	10	-0.972	0.388	0.015	3.1E-06
**VOD**	Lithium	40	7	0.101	0.048	0.043	1.8E-06
**WMD**	Lithium	40	7	-38	17	0.031	0.248
**WMN**	Lithium	40	9	-1043	273	3.5E-04	0.148
**WN**	Lithium	41	9	0.707	0.307	0.026	0.022
**WN**	Clioquinol	41	6	-0.998	0.304	0.002	0.027
**WSN**	Lithium	37	9	-0.021	0.008	0.008	0.847

abbreviations: N Control - number of untreated controls for specified intervention; N Intervention - number of animals treated with that intervention for specified assay; ß - mean change in outcome per 100 days over late life for the specified intervention relative to untreated controls; SE - standard error; P - P value; (*Phenotype*)D - average by day; (*Phenotype*)N - average by night; BG - fasted blood glucose level; EE.BM - energy expenditure/body mass; F - food consumed; PS - mean speed of walking; RQ - respiratory quotient; S - sleep; VC - VCO_2; VO - VO_2; W - water drunk; WM - wheel meters run; WS - wheel speed while running; P (sex interaction) $P < 0.05$ effect of intervention on ß was significantly different by sex.

Supplemental Table 34. Tortuosity measures kyphosis in 5-month old versus 19-month-old mice

Sex	n_total	ß	SE	P	n_5months	n_19months
M & F	122	0.026	0.005	7.1E-07	50	72
F	61	0.019	0.006	0.001	25	36
M	61	0.034	0.008	1.3E-04	25	36

abbreviations: M - Males; F - Females; n_total - total number of animals measured, ß - average difference in 19-months-old versus 5-months-old in tortuosity; SE - standard error of the mean; P - P value; n_5months - number of animals measured for tortuosity at 5-months-old; n_19months - number of animals measured for tortuosity at 19-months-old.

Supplemental Table 35. Doses used for each intervention

Intervention	Dosage in food
BS	2%
CQ	210 ppm
HBX	1ppm
Li_2CO_3	0.064%

Supplemental Table 36. Numbers of animals purchased and enrolled

Group	Male	Female	Age (mo)
**Young**	**25**	**25**	**5**
**BS**	**60**	**60**	**18**
**CQ**	**60**	**60**	**18**
**HBX**	**60**	**60**	**18**
**Li_2CO_3**	**60**	**60**	**18**
**Controls**	**120**	**120**	**18**

abbreviations: BS - Beta Sitosterol; CQ - Clioquinol; HBX - (2-(2-hydroxyphenyl) benzoxazole) ; Li_2CO_3

Supplemental Table 37. Femoral bone measures assayed

Phenotype	Units	Description
TV	mm^3	Total volume
BV	mm^3	Bone volume
BV.TV	%	Percent bone volume
TS	mm2	Tissue surface
TS.per.	%	Tissue surface (%)
BS	mm2	Bone surface
BS.per.	%	Bone surface percent
BS.BV	mm^{-1}	Bone surface/volume ratio
T.Ar	mm^2	Mean total crossectional tissue area
T.Pm	mm	Mean total crossectional tissue perimeter
B.Ar	mm^2	Mean total crossectional bone area
B.Pm	mm	Mean total crossectional bone perimeter
Ecc	Dimensionless number	Mean eccentricity
Cs.Th	mm	Crossectional thickness
Po.cl.	%	Closed porosity
endosteal	mm	endosteal perimeter
Med.area	mm^2	Medullary area
